# Genome-wide association studies for hematological traits in Chinese Sutai pigs

**DOI:** 10.1186/1471-2156-15-41

**Published:** 2014-03-27

**Authors:** Feng Zhang, Zhiyan Zhang, Xueming Yan, Hao Chen, Wanchang Zhang, Yuan Hong, Lusheng Huang

**Affiliations:** 1 Key Laboratory for Animal Biotechnology of Jiangxi Province and the Ministry of Agriculture of China, Jiangxi Agricultural University, 330045 Nanchang, China

**Keywords:** Single marker GWAS, Haplotype analysis, Hematological traits, Pig

## Abstract

**Background:**

It has been shown that hematological traits are strongly associated with the metabolism and the immune system in domestic pig. However, little is known about the genetic architecture of hematological traits. To identify quantitative trait loci (QTL) controlling hematological traits, we performed single marker Genome-wide association studies (GWAS) and haplotype analysis for 15 hematological traits in 495 Chinese Sutai pigs.

**Results:**

We identified 161 significant SNPs including 44 genome-wide significant SNPs associated with 11 hematological traits by single marker GWAS. Most of them were located on SSC2. Meanwhile, we detected 499 significant SNPs containing 154 genome-wide significant SNPs associated with 9 hematological traits by haplotype analysis. Most of the identified loci were located on SSC7 and SSC9.

**Conclusions:**

We detected 4 SNPs with pleiotropic effects on SSC2 by single marker GWAS and (or) on SSC7 by haplotype analysis. Furthermore, through checking the gene functional annotations, positions and their expression variation, we finally selected 7 genes as potential candidates. Specially, we found that three genes (*TRIM58, TRIM26* and *TRIM21*) of them originated from the same gene family and executed similar function of innate and adaptive immune. The findings will contribute to dissection the immune gene network, further identification of causative mutations underlying the identified QTLs and providing insights into the molecular basis of hematological trait in domestic pig.

## Background

Hematological cells play essential roles in the immune responding process of disease resistance [1,2]. Hematological cells are composed of three components, including leukocytes (white blood cells, WBCs), erythrocytes (red blood cells, RBCs) and platelets [3]. The major functions of leukocytes are innate and adaptive immunity and defending subject from pathogens [4,5]. White blood cell count is a strong indicator of infectious and inflammatory diseases, such as leukaemia and lymphoma. Erythrocytes execute a range of functions such as transporting oxygen, carbon dioxide and killing pathogens [6,7]. RBCs disorders indicate the increasing risk of anemia, polycythemia, hypertension and heart failure. Platelets play important roles in hemostasis, the initiation of wound repair and can be the strong effector cells of innate immune response [8-11]. Accompanying with reduction of platelet count, idiopathic thrombocytopenic purpura (ITP), often an idiopathic immune thrombocytopenia, may result in lower gastrointestinal bleeding or other internal bleeding in human [12]. Simply speaking, they are routinely measured as important clinical indicators to diagnose and monitor hematologic diseases and ascertain overall patient health condition.

The domestic pig is being increasingly exploited as an ideal model animal in human genetic diseases due to the high similarity with human physiological characteristics [13]. Therefore, discovering new loci for hematological traits and revealing their genetic mechanisms in domestic pig are conducive to the human blood disease. However, little is known about the association between genetic variation and hematological traits [14-17]. To our knowledge, 239 genome-wide significant quantitative trait loci (QTL) have been identified so far, which only explained a small fraction of the genetic variance (http://www.animalgenome.org/cgi-bin/QTLdb/SS/index) [18]. In these identified QTLs, the confidence intervals are generally large (> 20 cM) [19] and harbor thousands of functional genes, thereby hampering the identification of plausible candidate genes. Compared with traditional QTL mapping strategies, single marker GWAS [20,21] take advantage of linkage disequilibrium using high-density molecular markers rather than the linkage information using low-density markers in the intercross populations. Therefore, single marker GWAS could efficiently narrow down confidence interval of detected QTL and pick up the most associated markers for trait of interest. On the other hand, if the causative mutation is ancient, the LD between markers and mutated loci is too small to be captured with current marker density. Haplotype integrates linkage and linkage disequilibrium information [22] together, it is considered with the ability of overcoming the shortcoming in linkage and (or) single marker GWAS. Theoretically, haplotype analysis could acquire more accurate positions and shorter confidence intervals compared with separately performing linkage analysis or linkage disequilibrium analysis.

In this study, we conducted single marker GWAS and haplotype analysis of 15 hematological traits in Chinese Sutai population. The main purpose of the study is to reveal new loci associated with hematological trait and discover potential causative genes combining with biological and bioinformatics annotation. Furthermore, our result may also provide insights into the molecular basis of hematological trait in human.

## Results

### Phenotype statistics and SNP characteristics after quality control

The means, standard errors and coefficient of variation (C.V) of the phenotypic observations of the 15 hematological traits in the current experimental population are presented in Table 1. The C.V ranges from 3.73 to 38.71 as the minimum and maximum value for MCHC and PLT, respectively.

**Table 1:** Descriptive statistics of 15 hematological traits in the Sutai population

**Trait**	**Abbreviation**	**Value**^**1**^**(No.**^**2**^**)**	**C.V**^**3**^
Red blood cell count (10^12/L)	RBC	7.726 ± 0.974 (385)	12.61
Hemoglobin (g/L)	HGB	125.565 ± 12.178 (382)	9.7
Hematocrit (%)	HCT	0.426 ± 0.052 (385)	12.21
Mean corpuscular volume (fl)	MCV	55.25 ± 2.67 (385)	4.83
Mean corpuscular hemoglobin (pg)	MCH	16.387 ± 1.071 (384)	6.54
Mean corpuscular hemoglobin concentration (g/L)	MCHC	296.379 ± 11.069 (383)	3.73
Red cell distribution width (%)	RDW	17.501 ± 2.242 (317)	12.81
Red blood cell volume distribution width-SD(fL)	RDW-SD	34.841 ± 2.831 (384)	8.13
White blood cell count (10^9/L)	WBC	22.816 ± 5.43 (384)	23.8
Lymphocyte count (10^9/L)	LYM	10.519 ± 2.537 (378)	24.12
Lymphocyte count percentage (%)	LYMA	47.489 ± 10.12 (376)	21.31
Platelet count (10^9/L)	PLT	274.077 ± 106.084 (379)	38.71
Platelet-large cell ratio (%)	P-LCR	0.202 ± 0.05 (145)	24.75
Mean platelet volume (fl)	MPV	8.94 ± 0.641 (182)	7.17
Platelet distribution width (%)	PDW	13.2 ± 1.581 (182)	11.98

^1^Values are shown in mean ± standard deviation.

^2^The numbers of recorded individuals are given in parentheses.

^3^C.V: coefficient of variation.

After quality control, none of the individuals had a genotyping call rate < 95%, resulting in 495 individuals remained for the association analyses. Additionally, 3610 SNPs with call rates < 90%, 16242 SNPs with minor allele frequency (MAF) < 0.05, 64 SNPs severely departing from Hardy Weinberg Equilibrium (HWE) (P-value < 10^-5^) and 149 makers exhibiting Mendelian inconsistency were excluded, remained a total of 44650 SNPs. We also removed 4864 SNPs, including unmapped SNPs or SNPs on the sex chromosome. Finally, a total of 39786 SNPs were remained for further analyses.

### Erythrocyte traits

**Figure 1:**
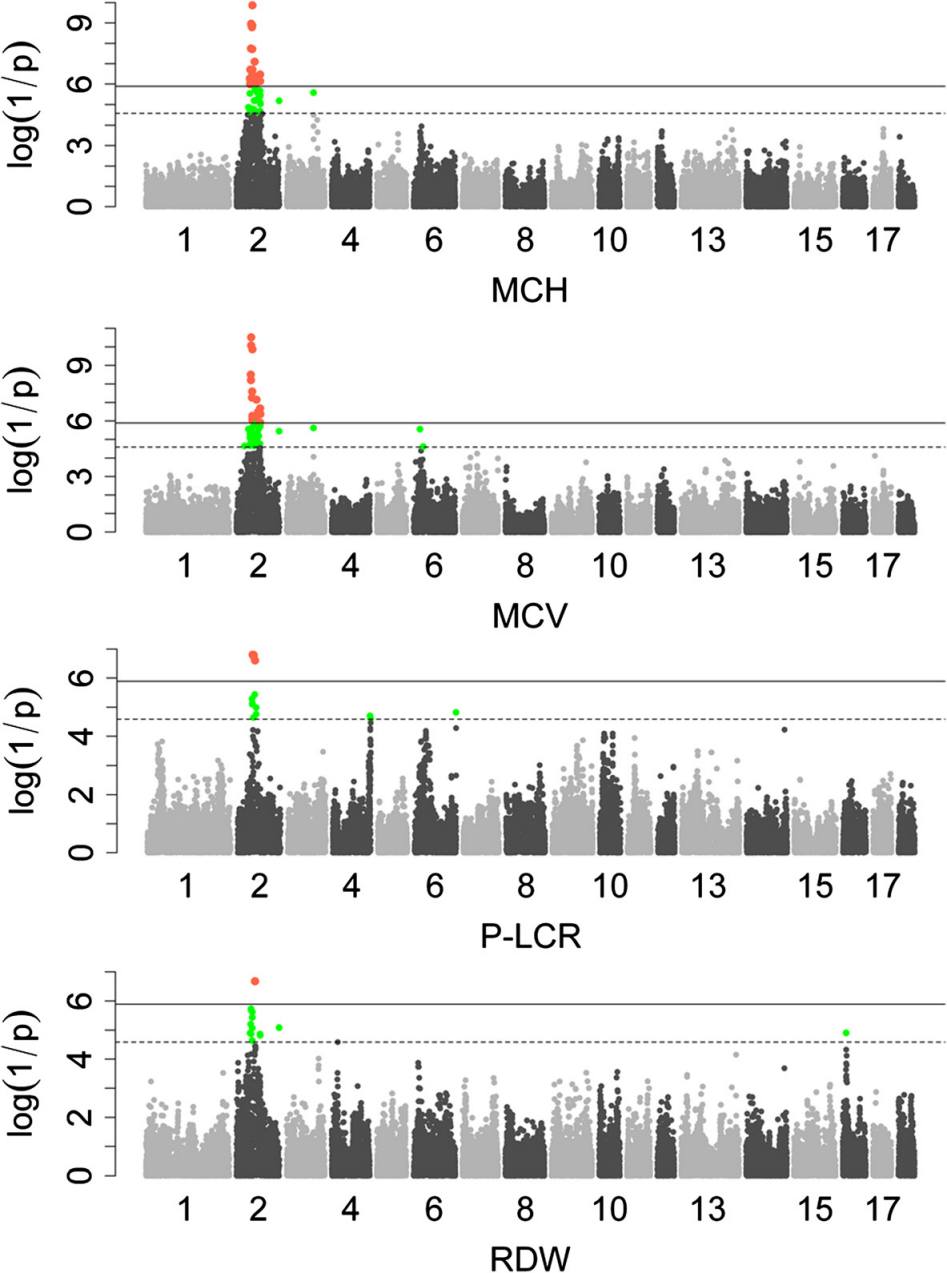
**Manhattan plots for the single marker analysis of hematological traits surpass genome-wide significant threshold.** log_10_(1/P-value) values are shown for all SNPs that passed quality control. The numbers indicate the chromosomes in the genome. The solid line and dotted line denotes the Bonferroni-corrected genome-wide and suggestive significant threshold, respectively. SNPs surpassing the genome-wide threshold are highlighted in pink and SNPs reaching the suggestive threshold in green. MCH: mean corpuscular hemoglobin; MCV: mean corpuscular volume; P-LCR: platelet-large cell ratio; RDW: red blood cell volume distribution width.

*Single marker GWAS*: In total, 141 significant SNPs (including 40 genome-wide and 101 suggestive SNPs) were identified for 8 erythrocyte traits: 5 for HCT, 1 for HGB, 40 for MCH, 22 for MCHC, 56 for MCV, 4 for RBC and 13 for RDW (Table 2 and Additional file 1: Table S1). All these 141 SNPs were located on SSC1, 2, 3, 4, 6,13 and 16; most of them located on SSC2 and SSC6 (Figure 1). No significant SNPs were detected for RDW-SD (Additional file 2: Figure S1). Eighty-three of the identified SNPs were located within 39 annotated genes, and 58 markers were located in region of 65 to 473458 bp away from the nearest annotated gene. In the 141 SNPs, 40 SNPs associated with at least two traits. They were mainly located on SSC2 and 13. And the most significant SNP (ss478944677) was associated with three erythrocyte traits: MCV (P-value =3.00 × 10^11^), MCH (P-value =1.10 × 10^9^) and RDW (P-value =1.86 × 10^6^).

**Table 2:** Description of lead SNPs showing significant association with hematological traits by GWAS

**Traits**^**1**^	**Peak SNP**	**Num**^**2**^	**Chr**^**3**^	**Pos (bp)**^**4**^	**Nearest gene**^**5**^	**Distance (bp)**^**6**^	**P-value**	**Var (%)**^**7**^
MCV^**^	ss478944677	75	2	55226096	SH3BP5L	Within	3.00E-11	19.41
MCH^**^	ss131190955	60	2	60227081	CPAMD8	Within	1.36E-10	7.91
RDW^**^	ss107831331	14	2	70061497	CARM1	Within	2.10E-07	14.13
MCHC^*^	ss131046473	22	6	49168322	ELSPBP1	65	1.52E-06	10.06
HCT^*^	ss131276048	5	4	104825883	INTS3	Within	3.19E-06	6.80
RBC^*^	ss131276048	4	4	104825883	INTS3	Within	3.78E-06	6.52
HGB^*^	ss131493776	1	13	11696883	UBE2E1	Within	7.88E-06	4.75
WBC^*^	ss107857076	2	2	105499649	ENSSSCG00000030166	95033	6.03E-06	8.66
P-LCR^**^	ss107886044	17	2	56469735	TRIM58	Within	1.58E-07	13.32
MPV^*^	ss107886044	5	2	56469735	TRIM58	Within	2.49E-06	15.43

The associated region was defined as the interval the distances between two adjacent genome-wide significant SNPs was less than 10 Mb.

^1^The abbreviations of hematological traits are given in Table 1. e.g. MCV is Mean corpuscular volume.

^2^The number of significant SNPs for each hematological trait.

^3, 4^Chromosomal locations and positions of the most significant SNP associated with hematological traits in Sus scrofa Build 10.2 assembly.

^5^The nearest annotated gene to the significant SNP. The annotated gene database is from http://asia.ensembl.org/index.html.

^6^SNP designated as in a gene or distance (bp) from a gene region in Sus scrofa Build 10.2 assembly, “0” in column 6 represent un-annotated genes.

^7^the phenotype variance explained by the significant SNP.

^**^Genome-wide significant.

^*^Chromosome-wide significant.

**Figure 2:**
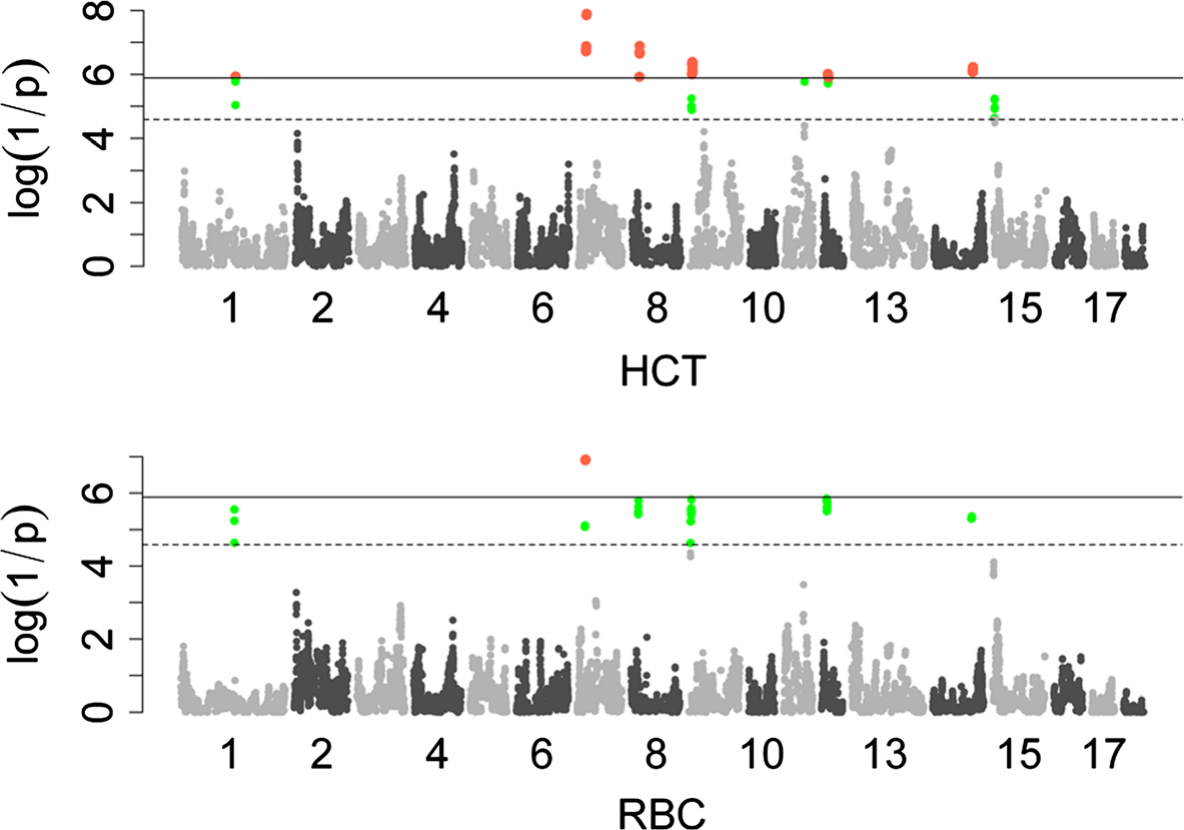
**Manhattan plots for the haplotype analysis of hematological traits surpass genome-wide significant threshold.** log_10_(1/P-value) values are shown for all SNPs that passed quality control. The numbers indicate the chromosomes in the genome. The solid line and dotted line denotes the Bonferroni-corrected genome-wide and suggestive significant threshold, respectively. SNPs surpassing the genome-wide threshold are highlighted in pink and SNPs reaching the suggestive threshold in green. HCT: hematocrit; RBC: red blood cell count.

*Haplotype analysis*: Totally, 498 significant SNPs (including 154 genome-wide and 344 suggestive SNPs) were identified for 8 erythrocyte traits: 192 for HCT, 60 for MCH, 68 for MCV, 165 for RBC and 13 for RDW-SD (Table 3 and Additional file 3: Table S2). These significant SNPs were located on SSC1, 2, 4, 5, 7, 8, 9, 11, 12, 14 and 15 and most of them were located on SSC7 and 9 (Figure 2). No significant SNPs were detected in association with HGB, MCH and RDW (Additional file 4: Figure S2). The top SNP ss107842725 located in ENSSSCG00000001232 gene on SSC7 was associated with HCT, RBC and MCV. Furthermore, 38 of 154 genome-wide significant SNPs were located within the regions of 24 annotated genes and the others were located in the regions of the nearest known genes with the distance from 62 to 757213 bp.

**Table 3:** Description of lead SNPs showing significant association with hematological traits by LDLA

**Traits**^**1**^	**Peak SNP**	**Num**^**2**^	**Chr**^**3**^	**Pos (bp)**^**4**^	**Nearest gene**^**5**^	**Distance (bp)**^**6**^	**P-value**
HCT^**^	ss107842725	331	7	24777963	ENSSSCG00000001232	Within	1.28E-08
RBC^**^	ss107842725	180	7	24777963	ENSSSCG00000001232	Within	1.20E-07
MCH^*^	ss478941323	60	5	61925570	GRIN2B	144801	3.05E-06
MCV^*^	ss107842725	68	7	24777963	ENSSSCG00000001232	Within	1.47E-06
RDW-SD^*^	ss131114130	13	9	33331814	ENSSSCG00000014974	3311	1.89E-05
WBC^*^	ss131152863	1	1	289943447	TLR4	157600	1.49E-05

The associated region was defined as the interval the distances between two adjacent genome-wide significant SNPs was less than 10 Mb.

^1^The abbreviations of hematological traits are given in Table 1. e.g. MCV is Mean corpuscular volume.

^2^The number of significant SNPs for each hematological trait.

^3, 4^Chromosomal locations and positions of the most significant SNP associated with hematological traits in Sus scrofa Build 10.2 assembly.

^5^The nearest annotated gene to the significant SNP. The annotated gene database is from http://asia.ensembl.org/index.html.

^6^SNP designated as in a gene or distance (bp) from a gene region in Sus scrofa Build 10.2 assembly, “0” in column 6 represent un-annotated genes.

^**^Genome-wide significant.

^*^Chromosome-wide significant.

### White blood cell counts

*Single marker GWAS*: Analysis of white blood cell counts revealed two significant loci on SSC2 by single marker GWAS. The most significant SNP ss107857076 (P-value = 6.03 × 10^-6^) associated with WBC was located at 105499649 bp on SSC2 with a distance of 95033 bp away from ENSSSCG00000030166 gene. The remaining SNP ss131195511 was located at 101149437 bp on SSC2 and 277118 bp away from gene *GPR98* (G protein-coupled receptor 98).

*Haplotype analysis*: One significant locus associated with WBC was identified by haplotype analysis. The SNP ss131152863 was located at 289943447 bp on SSC1 and 157600 bp away from *TLR4* (toll-like receptor 4) gene.

### Platelet traits

*Single marker GWAS*: Eighteen SNPs significantly associated with two platelet traits were detected by single marker GWAS: 13 for P-LCR and 5 for MPV. They were located on SSC2 and distributed within a 10.7 Mb region (54474152–65200938 bp). Both P-LCR and MPV shared the same top SNP of ss107886044 which was located in an annotated gene *TRIM58* (tripartite motif containing 58) at 105499649 bp.

*Haplotype analysis*: No significant SNP was detected by haplotype analysis.

## Discussion

The Sutai pigs were generated by intercross of Meishan (Erhualian) female and Duroc male for about 25 generations. Their genome was composed of a mosaic of small pieces of haplotype segments derived from both breed. As a result, their LD block was much smaller than classic QTL mapping populations [23]. Sutai pigs included two kinds of LD: LD between breeds created by intercross and LD within each breed created in the ancestor history, and they hence become very good experimental population for QTL mapping and single marker GWAS analysis.

### Comparison with previous studies

By performing single marker GWAS and haplotype analyses, we identified 651 SNPs associated with the 15 hematologic traits. Of these SNPs, 253 located within known genes’ region, 265 located near to the annotated gene and 133 weren’t mapped to the current assembled genome (Sus Scrofa Build 10.2, http://asia.ensembl.org/index.html). So far, several papers reported single marker GWAS result for hematological traits in pig. Zhang *et al.* using similar association strategy revealed 185 genome-wide significant SNPs for 18 hematological traits in 1020 white Duroc x Erhualian F2 intercross [24]. Most of the identified significant SNPs were located on SSC8. Luo *et al.*[25] detected 62 genome-wide significant and 3 chromosome-wide significant SNPs associated with erythrocyte traits on a Large White × Chinese Min F2 intercross and most of them also retained on SSC8. Both of them pinpoint that *KIT* (v-kit Hardy-Zuckerman 4 feline sarcoma viral oncogene homolog) gene as the potential candidate. In our study, we didn’t detect any signal associated with erythrocyte traits in this region. *KIT* is essential for coat color while all individuals’ in our study is black. Hence there was no variation at *KIT* gene and of course without association signal. None significant SNP in Luo *et al.* and Zhang *et al.* was overlapped with our study. The reasons for the inconsistence by similar analysis strategy could be monomorphic at the causative locus, the population heterogeneity and the complex genetic background. These results also hint that hematological traits was a complex trait which affected by multiple genes. Wang *et al.*[26] identified 111 significant SNPs for 18 hematological traits after injected classical fever vaccine in 2 Western breeds and one Chinese synthetic breed by similar single marker association study. Their mapping result might include both QTL affecting immune responses and QTL affecting base hematological traits. Herein we found 9 SNPs on SSC6 were identical with the results of our present study, while none functional gene was posited in that region.

### Comparison between single marker GWAS and haplotype analysis results

In this study, we performed both single marker GWAS and haplotype analysis to explore potential causal gene(s) for hematological traits in Chinese Sutai pigs. Only 9 SNPs located on SSC2 were overlapped by both analyses for MCH. The basic principle of single marker GWAS was to compare phenotypic differences grouped by alleles. If the marker density was not high enough, the significant SNPs may lose because of low LD between markers and causative mutation. However, haplotype will surmount this disadvantage. Druet and Georges [27] have fully descripted haplotype analysis, which took advantage of recent and ancestral recombination events simultaneously. In here, we used haplotype analysis and identified 490 SNPs located on SSC1, 2, 4, 5, 7, 8, 9, 11, 12, 14 and 15 which can’t be detected by single marker GWAS. However, one drawback of haplotype analysis is the reduction of detection power, because its degree of freedom is generally bigger than single marker analysis. Zhang *et al.*[28] also pinpoint this phenomenon due to the degree of freedom. However, the balance between increasing LD and decreasing power by the degree of freedom is hard to weight. Moreover, LD across whole genome is inhomogeneous – there are high LD in some regions and low LD in other regions. In this case, we recommend performing both single marker and haplotype analyses strategies to capture more associated SNPs. We obtained 141 significant SNPs by single marker analysis and 498 SNPs by haplotype analyses for 8 erythrocyte traits. In together, 651 significant were identified associating with hematological traits, which was more than any one analyses strategy.

### Possible pleiotropic QTLs

The patterns of Manhattan plots of MCH, MCV and P-LCR were similar, and they shared a common region ranging from 54.47 Mb to 55.24 Mb containing three SNPs (ss131191392, ss478944677 and ss131085967) on SSC2. MCH and MCV are parameters reflecting average weight of hemoglobin per RBC and average volume of RBC, respectively. By analysis of the correlation among the hematological traits (Additional file 5: Table S3), high correlation between the two traits was observed (r = 0.804, P-value < 1.0 × 10^-16^). This result implied the QTL on SSC2 might be pleiotropic. Marker ss107842725, located at 24777963 bp on SSC7, was the top SNP associated with HCT, MCH and RBC. The Manhattan plot also explored very similar patterns for the three phenotypes. HCT, MCH and RBC mainly measure fluctuation of red blood cell and they may segregate dependently. Our results indicated that pleiotropic QTL was common on hematological traits. In clinical diagnosis, the three parameters (HCT, MCH and RBC) could be integrated together for more precisely diagnose.

### Potential candidate functional genes

In total, we identified 161 significant SNPs on 7 different chromosomes associated with hematological traits by single marker GWAS (Additional file 1: Table S1). Among these SNPs, 25 SNPs were found within 14 annotated genes from 52.14 to 90.17 Mb. Through checking these annotated gene functions, we eventually selected four genes as potential candidate genes. The four genes, *TRIM58, CPAMD8* (C3 and PZP-like, alpha-2-macroglobulin domain containing 8)*, ABCA7* (ATP-binding cassette, sub-family A (ABC1), member 7) *and JAK3* (Janus kinase 3), were functionally associated with hematological related cells or immune function.

The SNP ss107886044 located in *TRIM58* gene explained 15.43% (Table 2) of phenotypic variants of P-LCR. Christopher *et al.* regarded *TRIM58* as an E3 ubiquitin ligase that regulated terminal erythroid cell cycles and enucleation [29]. Moreover, the *TRIM58* protein was involved in pathogen-recognition [30] and the regulation of innate immune responses [31]. Therefore, the *TRIM58* gene could be regarded as a strong candidate gene controlling P-LCR. In addition to *TRIM58*, the SNP marker (ss131190955) within the *CPAMD8* gene also showed high association signal with a P-value of 1.33 × 10^-10^. The *CPAMD8* gene was highly conserved, which may have similar function like other members of the C3/α_2_M family and also be involved in innate immunity [32-34]. A promising gene *ABCA7* was a member of the super family of ATP-binding cassette (ABC) transporters, expression of which was induced during vitro differentiation of human monocytes into macrophages. Besides, *ABCA7* mRNA was detected predominantly in myelo-lymphatic tissues with highest expression in peripheral leukocytes [35,36]. *JAK3* was predominantly expressed in hematopoietic cells, such as NK cells, T cells and B cells [37] and transduced a signal in response to its activation. Furthermore, mutations which abrogated *JAK3* might cause an autosomal SCID (severe combined immunodeficiency disease) [38].

By haplotype analysis, we identified 154 genome-wide significantly loci mainly SSC7 and SSC9. Among them, 50 significant SNPs for HCT were found within 34 annotated genes and 4 significant SNPs for RBC were found within 4 annotated genes. In these annotated genes, three genes *TRIM26* on SSC7, *TRIM21* (tripartite motif containing 21) and *NUP98* (nucleoporin 98 kDa) on SSC9 were picked up as potential candidates by checking their gene functions. These genes were functionally associated with hematological related cells or immune function.

The *TRIM26*, encoding a member of the tripartite motif (TRIM) family, was located within the SLA region [39]. Lee *et al.* also speculated that the *TRIM26* gene played essential roles in the human immune response because of its predicted protein function [40]. In addition to *TRIM26*, the *TRIM21* gene also belonged to the tripartite motif (TRIM) family. It was an E3 ubiquitin ligase for IFN regulatory factor IRF3 and IRF8 with the function of innate and adaptive immunity [41]. Yang *et al.* demonstrated that *TRIM21* interacts with PIN1 mediates the ubiquitination and degradation of IRF3 during virus infection [42]. Besides, it was reported that *TRIM21* may regulate T-cell activation or proliferation, since overexpression of *TRIM21* had been shown to increase IL-2 production in CD28-stimulated Jurkat T cells [43]. Therefore we could regard the *TRIM21* gene, involved in both physiological immune responses and pathological autoimmune processes [44], as a strong candidate gene. The *NUP98* fusion proteins had been shown to inhibit differentiation of hematopoietic precursors and to increase self-renewal of hematopoietic stem or progenitor cells [45]. The *NUP98* gene also was known to be fused to at least 28 different partner genes in patients with hematopoietic malignancies, including acute myeloid leukemia, chronic myeloid leukemia in blast crisis, myelodysplastic syndrome, acute lymphoblastic leukemia, and bilineage/biphenotypic leukemia.

In all identified genes, we specially pointed out three genes (*TRIM58*, *TRIM26* and *TRIM21*), which belonged to the same gene family. The three genes executed similar function of innate and adaptive immune and communicated together in the immune network system. Our result revealed a series of key driver genes in the immune network system.

## Conclusions

In summary, we identified 651 SNPs, some of which were pleiotropic. Such as three SNPs on SSC2 associated with MCV, MCH and P-LCR and ss107842725 on SSC7 associated with HCT, MCH and RBC. What’s more, we selected 7 genes as potential candidates based on their functional annotations, positions and reported expression variation. Especially, three strong candidate genes (*TRIM58*, *TRIM26* and *TRIM21*) may be the key driver genes in the immune network system. These findings will conduct further studies to examine the identified SNPs in other diverse population and pursue functional validation for identification of the causal mutation.

## Methods

### Ethics statement

All procedures involving animals followed the guidelines for the care and use of experimental animals approved by the State Council of the People’s Republic of China.

### Study populations and phenotype measurement

The Sutai population comprised with 436 offspring of 4 boars and 55 sows. Each boar mated with 13 to 15 sows to make the family structure in balance. There were three batches of piglets which were almost born in three different months (April, June and July, 2011) at Sutai Pig Breeding Center in Suzhou city. At the age of 2–3 month, then the piglets were transferred to a farm in Nanchang city. All Sutai piglets were castrated and weaned at 18 days and 28 days after birth, respectively. They were fed with same diet (formulated according to age) under a standardized feeding and management regimen, and given free access to water. At 240 ± 6 days of age, a total of 436 Sutai offspring including 206 gilts and 230 barrows were slaughtered at a commercial abattoir.

Blood samples of 5 ml were immediately collected from each animal when it was slaughtered and directly injected into eppendorf tubes containing 30 ul of 20% EDTA in polybutadiene-styrene. A standard set of hematological data were recorded using a CD1700 whole blood analyzer (Abbott, USA) in 24 h postmortem at the First Affiliated Hospital of NanChang University, China. Fifteen hematological parameters including 8 baseline erythroid traits (hematocrit (HCT), hemoglobin (HGB), mean corpuscular hemoglobin (MCH), mean corpuscular hemoglobin concentration (MCHC), mean corpuscular volume (MCV), red blood cell count (RBC), red blood cell volume distribution width-SD (RDW-SD), and red blood cell volume distribution width (RDW)), 3 leukocyte traits (lymphocyte count (LYM), lymphocyte count percentage (LYMA), and white blood cell count (WBC)), and 4 platelet traits (platelet distribution width (PDW), platelet count (PLT), platelet-large cell ratio (P-LCR) and mean platelet volume (MPV)) were used for performing single marker GWAS. The correlations between 15 hematological parameters were performed by R psych package (http://personality-project.org/r/psych.manual.pdf).

### Genotyping and quality control

Genomic DNA was extracted from ear tissues using a standard phenol/chloroform method [46]. All DNA samples were qualified and standardized into a final concentration of 50 ng/ul. A total of 436 offspring and their 59 parents in the Sutai pedigree were genotyped for the Porcine SNP60 Beadchips on an iScan System (Illumina, USA) following the manufacturer’s protocol. Quality control was carried out using PLINK (version 1.07) [47] and executed to exclude SNPs with parameter of call rate < 90%, minor allele frequency (MAF) < 5%, severely departed from HWE (P-value < 10^-5^) and Mendelian inconsistency rate > 10%. Moreover, individuals with missing genotypes > 10% or Mendelian errors > 5% were discarded for further analysis.

### Statistical analyses

The genome-wide and suggestive significance thresholds in the two association strategies were determined by the Bonferroni correction, in which the conventional P-value was divided by the number of tests performed [48]. A SNP was considered to have genome-wide significance at P-value < 0.05/N and have suggestive significance at P-value < 1/N, where N is the number of SNPs tested in the analyses. The corresponding thresholds were set as 1.26 × 10^-6^ (0.05/39786) and 2.51 × 10^-5^ (1/39786) in this study.

#### Single marker GWAS

The linear tendency of allelic and phenotypic traits was tested using a general linear mixed model for each SNP [49-51]. The model included a random polygenic effect and the variance-covariance matrix was proportionate to genome-wide identity-by-state [52]. The model was described as following: *Y* = *Xb* + S*α* + *Zu* + *e*, where Y is the vector of phenotypes, b is the estimator of fixed effects including sex and batch, *α* is the SNP substitution effect and u is the random additive genetic effect following multinomial distribution u ~ N (0, Gσ_α_^2^), in here G is the genomic similarity matrix that was constructed based on SNP markers as described in Eding *et al.*[53], and σ_α_^2^ is the polygenetic additive variance. X, S and Z are the incidence matrices for b, *α* and u, respectively. *e* is a vector of residual errors with a distribution of N (0, Iσ_e_^2^). The single marker GWAS were conducted by GenABEL packages in the R software [54,55].

#### Haplotype analysis

The haplotypes were constructed following Druet & Georges, by using a Hidden Markov Model via PHASEBOOK [27] that assumes the existence of a predetermined number of ancestral haplotype states (K = 20) from which all haplotypes in the population are derived [56]. The statistical model used for the haplotype analysis was identical to that of single marker GWAS except that a haplotype effect was fitted instead of a SNP effect [57]. The haplotype was followed approximately the approach of Meuwissen and Goddard [22,31,58,59], except that haplotypes were assumed to be completely uncorrelated, instead of fitting a more differentiating identity by descent (IBD) matrix G.

#### Phenotypic variants analysis

The fraction of phenotype variances explained by detected SNP was computed by following formula:

$$\mathrm{Var\%}=\frac{\left(MS_{\mathrm{reduce}1}-MS_{\mathrm{full}}\right)}{MS_{\mathrm{reduce}}}\times 100$$

Where *MS*_*full*_*, MS*_*reduce1*_*and MS*_*reduce*_ were the mean square (MS) in the linear models including three effects (mean, sex and SNP), including two effects (mean and sex) and only including mean, respectively.

## Competing interests

The authors declare that they have no competing interests.

## Authors’ contributions

LH conceived and led the coordination of the study. ZZ and FZ led the data analysis and the manuscript preparation. XY, WZ, HC and YH contributed to blood collection and slaughter. FZ, WZ, HC and YH directed the genotyping work and recording hematological data. XY, ZZ and FZ interpreted the results and contributed to edit the manuscript. All authors read and approved the final manuscript.

## Authors’ information

Feng Zhang and Zhiyan Zhang are co-first authors.

## Supplementary Material

Additional file 1: Table S1 Description of all identified SNPs showing significant association with hematological traits by single marker GWAS. Click here for file

Additional file 2: Figure S1 Manhattan plots for the single marker analysis of hematological traits surpass suggestive significant threshold. log_10_ (1/P-value) values are shown for all SNPs that passed quality control. The solid line and dotted line denotes the Bonferroni-corrected genome-wide and suggestive significant threshold respectively. SNPs reaching the suggestive threshold are highlighted in green. HCT: hematocrit; HGB: hemoglobin; MCHC: mean corpuscular hemoglobin content; RBC: red blood cell; WBC: white blood cell count; MPV: mean platelet volume. Click here for file

Additional file 3: Table S2 Description of all identified SNPs showing significant association with hematological traits by haplotype analysis. Click here for file

Additional file 4: Figure S2 Manhattan plots for the haplotype analysis of hematological traits surpass suggestive significant threshold. log_10_ (1/P-value) values are shown for all SNPs that passed quality control. The solid line and dotted line denotes the Bonferroni-corrected genome-wide and suggestive significant threshold respectively. SNPs reaching the suggestive threshold are highlighted in green. MCH: mean corpuscular hemoglobin; MCV: mean corpuscular volume; RDW-SD: red blood cell volume distribution width-SD; WBC: white blood cell count. Click here for file

Additional file 5: Table S3 Description of correlation and P-value among the 15 hematological traits. Click here for file
