# Supplementary material for: Genome-wide association studies for hematological traits in Chinese Sutai pigs
Source: BMC Genet. 2014 Mar 27;15:41. doi: 10.1186/1471-2156-15-41 (PMC3986688; doi:10.1186/1471-2156-15-41)
Supplement: Additional file 1: Table S1 — Description of all identified SNPs showing significant association with hematological traits by single marker GWAS. [file 1471-2156-15-41-S1.docx]

**Table S1 Description of all identified SNPs showing significant association with hematological traits by single marker GWAS**

| Traits^1^ | SNP name | Chr^2^ | Pos(bp)^3^ | Nearest gene^4^ | Distance（bp)^5^ | P-value |
| --- | --- | --- | --- | --- | --- | --- |
| HCT | ss131276048 | 4 | 104825883 | INTS3 | within | 3.19E-06 |
| HCT | ss131176755 | 1 | 98564209 | IRAK1BP1 | 473458 | 1.07E-05 |
| HCT | ss478937610 | 4 | 109288769 | 0 | within | 1.21E-05 |
| HCT | ss131206352 | 2 | 146082066 | KDM3B | within | 1.22E-05 |
| HCT | ss131493776 | 13 | 11696883 | UBE2E1 | within | 1.86E-05 |
| HGB | ss131493776 | 13 | 11696883 | UBE2E1 | within | 7.88E-06 |
| MCH | ss131190955 | 2 | 60227081 | CPAMD8 | within | 1.36E-10 |
| MCH | ss478944677 | 2 | 55226096 | SH3BP5L | within | 1.10E-09 |
| MCH | ss131085967 | 2 | 55240133 | SH3BP5L | 2589 | 1.24E-09 |
| MCH | ss478938204 | 2 | 59447736 | NIS | within | 1.30E-09 |
| MCH | ss107845661 | 2 | 58377827 | PBX4 | 4973 | 1.59E-09 |
| MCH | ss131191392 | 2 | 54474152 | JMJD4 | 54026 | 1.78E-08 |
| MCH | ss478940493 | 2 | 59489740 | JAK3 | within | 1.90E-08 |
| MCH | ss131089274 | 2 | 68396199 | 0 | 3576 | 7.97E-08 |
| MCH | ss131190969 | 2 | 60202374 | CPAMD8 | within | 1.93E-07 |
| MCH | ss131057482 | 2 | 52143852 | MRVI1 | within | 1.94E-07 |
| MCH | ss131190939 | 2 | 60261433 | CPAMD8 | within | 3.23E-07 |
| MCH | ss131193475 | 2 | 89541402 | 0 | within | 3.34E-07 |
| MCH | ss131189210 | 2 | 50640588 | DKK3 | 59397 | 5.20E-07 |
| MCH | ss131100103 | 2 | 75900120 | NCLN | within | 5.56E-07 |
| MCH | ss131189272 | 2 | 51244889 | 7SK | 42096 | 5.57E-07 |
| MCH | ss131193147 | 2 | 89042043 | 0 | 6910 | 7.24E-07 |
| MCH | ss107884309 | 2 | 53681830 | 0 | 8852 | 7.82E-07 |
| MCH | ss131190995 | 2 | 59957369 | HAUS8 | within | 8.72E-07 |
| MCH | ss478944893 | 2 | 51697818 | GALNTL4 | 187259 | 1.02E-06 |
| MCH | ss478937495 | 2 | 70246409 | SMARCA4 | within | 1.16E-06 |
| MCH | ss131189769 | 2 | 70506577 | ZNF653 | within | 1.88E-06 |
| MCH | ss131193319 | 2 | 89162092 | 0 | 108133 | 2.13E-06 |
| MCH | ss131084475 | 2 | 77635095 | ABCA7 | within | 2.59E-06 |
| MCH | ss131223663 | 3 | 102371501 | 0 | 14729 | 2.67E-06 |
| MCH | ss131034821 | 2 | 50604043 | 0 | 48573 | 2.95E-06 |
| MCH | ss131193744 | 2 | 90104938 | PAPD4 | 23314 | 3.53E-06 |
| MCH | ss478937548 | 2 | 89593299 | 0 | within | 4.10E-06 |
| MCH | ss478940501 | 2 | 82680942 | UNC5A | within | 5.11E-06 |
| MCH | ss131191898 | 2 | 84866415 | 0 | 118584 | 6.17E-06 |
| MCH | ss120020604 | 2 | 66776495 | ZNF791 | 306 | 6.46E-06 |
| MCH | ss107875173 | 2 | 161857853 | 0 | 10416 | 6.59E-06 |
| MCH | ss131189821 | 2 | 69458456 | ICAM1 | within | 6.68E-06 |
| MCH | ss478937928 | 2 | 90176527 | PAPD4 | within | 8.13E-06 |
| MCH | ss131567467 | 2 | 90675248 | SERINC5 | within | 9.58E-06 |
| MCH | ss131187906 | 2 | 45331023 | PLEKHA7 | within | 1.38E-05 |
| MCH | ss478944895 | 2 | 51697485 | GALNTL4 | 186926 | 1.54E-05 |
| MCH | ss131191283 | 2 | 57506678 | OR2G2 | 28402 | 1.64E-05 |
| MCH | ss131190211 | 2 | 66138044 | CACNA1A | within | 1.74E-05 |
| MCH | ss131193779 | 2 | 90190570 | CMYA5 | 3579 | 2.16E-05 |
| MCH | ss131189234 | 2 | 51376792 | GALNTL4 | within | 2.16E-05 |
| MCHC | ss131046473 | 6 | 49168322 | ELSPBP1 | 65 | 1.52E-06 |
| MCHC | ss131320622 | 6 | 50638891 | IL4I1 | within | 4.32E-06 |
| MCHC | ss478938382 | 6 | 46250517 | CADM4 | within | 5.54E-06 |
| MCHC | ss107873190 | 6 | 49817264 | RASIP1 | within | 6.38E-06 |
| MCHC | ss107904366 | 6 | 49351202 | ZNF114 | 3994 | 9.37E-06 |
| MCHC | ss478938384 | 6 | 47573330 | CCDC61 | within | 1.00E-05 |
| MCHC | ss131042465 | 6 | 47852634 | GNG8 | 1880 | 1.00E-05 |
| MCHC | ss131084466 | 6 | 48717238 | ZNF541 | within | 1.00E-05 |
| MCHC | ss131037385 | 6 | 49146524 | BSPH1 | within | 1.00E-05 |
| MCHC | ss131068147 | 6 | 45510367 | MEGF8 | within | 1.14E-05 |
| MCHC | ss478936181 | 6 | 45563650 | 0 | 4976 | 1.14E-05 |
| MCHC | ss131119887 | 6 | 45580300 | ERF | 16764 | 1.14E-05 |
| MCHC | ss131078268 | 6 | 45610428 | GSK3A | within | 1.14E-05 |
| MCHC | ss478937461 | 6 | 45617812 | GSK3A | within | 1.14E-05 |
| MCHC | ss131088045 | 6 | 46003088 | CXCL17 | within | 1.14E-05 |
| MCHC | ss131117283 | 6 | 46270959 | CADM4 | within | 1.14E-05 |
| MCHC | ss131320598 | 6 | 50532885 | AP2A1 | within | 1.30E-05 |
| MCHC | ss478936371 | 6 | 49802217 | MAMSTR | within | 1.33E-05 |
| MCHC | ss107815866 | 6 | 44231753 | U6 | 26380 | 1.33E-05 |
| MCHC | ss131047103 | 6 | 45508033 | MEGF8 | within | 1.36E-05 |
| MCHC | ss107816345 | 6 | 45609490 | GSK3A | within | 1.67E-05 |
| MCHC | ss131055010 | 6 | 48031641 | SLC1A5 | within | 1.98E-05 |
| MCV | ss478944677 | 2 | 55226096 | SH3BP5L | within | 3.00E-11 |
| MCV | ss131085967 | 2 | 55240133 | SH3BP5L | 2589 | 8.43E-11 |
| MCV | ss131190955 | 2 | 60227081 | CPAMD8 | within | 1.33E-10 |
| MCV | ss107884309 | 2 | 53681830 | 0 | 8852 | 3.16E-09 |
| MCV | ss131191392 | 2 | 54474152 | JMJD4 | 54026 | 6.26E-09 |
| MCV | ss478938204 | 2 | 59447736 | NIS | within | 2.57E-08 |
| MCV | ss107845661 | 2 | 58377827 | PBX4 | 4973 | 5.44E-08 |
| MCV | ss131100103 | 2 | 75900120 | NCLN | within | 7.07E-08 |
| MCV | ss478937548 | 2 | 89593299 | 0 | within | 2.08E-07 |
| MCV | ss478940501 | 2 | 82680942 | UNC5A | within | 3.09E-07 |
| MCV | ss478937928 | 2 | 90176527 | PAPD4 | within | 4.27E-07 |
| MCV | ss131084475 | 2 | 77635095 | ABCA7 | within | 4.77E-07 |
| MCV | ss131190995 | 2 | 59957369 | HAUS8 | within | 5.32E-07 |
| MCV | ss131190939 | 2 | 60261433 | CPAMD8 | within | 5.85E-07 |
| MCV | ss131089274 | 2 | 68396199 | 0 | 3576 | 8.25E-07 |
| MCV | ss131190969 | 2 | 60202374 | CPAMD8 | within | 8.88E-07 |
| MCV | ss478937495 | 2 | 70246409 | SMARCA4 | within | 1.05E-06 |
| MCV | ss131189601 | 2 | 74669571 | UHRF1 | 9213 | 1.08E-06 |
| MCV | ss131193475 | 2 | 89541402 | 0 | within | 1.23E-06 |
| MCV | ss131189617 | 2 | 74701437 | UHRF1 | within | 1.43E-06 |
| MCV | ss131189769 | 2 | 70506577 | ZNF653 | within | 1.47E-06 |
| MCV | ss131096150 | 2 | 71990984 | FCER2 | within | 1.55E-06 |
| MCV | ss131193147 | 2 | 89042043 | 0 | 6910 | 1.64E-06 |
| MCV | ss131193744 | 2 | 90104938 | PAPD4 | 23314 | 2.01E-06 |
| MCV | ss478940493 | 2 | 59489740 | JAK3 | within | 2.22E-06 |
| MCV | ss131223663 | 3 | 102371501 | 0 | 14729 | 2.43E-06 |
| MCV | ss120020052 | 2 | 84850085 | 0 | 102254 | 2.65E-06 |
| MCV | ss107866053 | 6 | 24389455 | U6 | 371565 | 2.80E-06 |
| MCV | ss131187906 | 2 | 45331023 | PLEKHA7 | within | 2.83E-06 |
| MCV | ss131189210 | 2 | 50640588 | DKK3 | 59397 | 3.04E-06 |
| MCV | ss107875173 | 2 | 161857853 | 0 | 10416 | 3.57E-06 |
| MCV | ss107831331 | 2 | 70061497 | CARM1 | within | 4.64E-06 |
| MCV | ss131191430 | 2 | 80262803 | TBC1D9B | within | 4.74E-06 |
| MCV | ss131057482 | 2 | 52143852 | MRVI1 | within | 5.06E-06 |
| MCV | ss131034821 | 2 | 50604043 | 0 | 48573 | 5.13E-06 |
| MCV | ss131191898 | 2 | 84866415 | 0 | 118584 | 6.39E-06 |
| MCV | ss107849200 | 2 | 72266697 | INSR | 8996 | 6.67E-06 |
| MCV | ss131189888 | 2 | 69240930 | 0 | within | 7.47E-06 |
| MCV | ss131189272 | 2 | 51244889 | 7SK | 42096 | 8.11E-06 |
| MCV | ss131564910 | 2 | 64970665 | CLEC17A | 92338 | 8.23E-06 |
| MCV | ss131056081 | 2 | 65057680 | CLEC17A | 5323 | 8.23E-06 |
| MCV | ss131191486 | 2 | 80486384 | CANX | 3875 | 8.59E-06 |
| MCV | ss131189626 | 2 | 74624436 | 0 | within | 1.00E-05 |
| MCV | ss131099546 | 2 | 65200938 | 0 | within | 1.09E-05 |
| MCV | ss107886044 | 2 | 56469735 | TRIM58 | within | 1.23E-05 |
| MCV | ss131189703 | 2 | 72633349 | 0 | 1455 | 1.32E-05 |
| MCV | ss131190872 | 2 | 60338816 | SIN3B | within | 1.34E-05 |
| MCV | ss131033234 | 2 | 55304153 | 0 | 42870 | 1.44E-05 |
| MCV | ss131190887 | 2 | 60318677 | SIN3B | within | 1.60E-05 |
| MCV | ss131193319 | 2 | 89162092 | 0 | 108133 | 1.74E-05 |
| MCV | ss131189234 | 2 | 51376792 | GALNTL4 | within | 1.85E-05 |
| MCV | ss131189821 | 2 | 69458456 | ICAM1 | within | 1.94E-05 |
| MCV | ss478944893 | 2 | 51697818 | GALNTL4 | 187259 | 2.19E-05 |
| MCV | ss120020604 | 2 | 66776495 | ZNF791 | 306 | 2.20E-05 |
| MCV | ss131089873 | 2 | 31120539 | 0 | within | 2.26E-05 |
| MCV | ss131191504 | 2 | 80670019 | ADAMTS2 | 79825 | 2.26E-05 |
| MPV | ss107886044 | 2 | 56469735 | TRIM58 | within | 2.49E-06 |
| MPV | ss131190887 | 2 | 60318677 | SIN3B | within | 2.55E-06 |
| MPV | ss131190872 | 2 | 60338816 | SIN3B | within | 2.99E-06 |
| MPV | ss131099546 | 2 | 65200938 | 0 | within | 6.57E-06 |
| MPV | ss131191392 | 2 | 54474152 | JMJD4 | 54026 | 1.53E-05 |
| P-LCR | ss107886044 | 2 | 56469735 | TRIM58 | within | 1.58E-07 |
| P-LCR | ss131190887 | 2 | 60318677 | SIN3B | within | 1.60E-07 |
| P-LCR | ss131190872 | 2 | 60338816 | SIN3B | within | 1.79E-07 |
| P-LCR | ss131099546 | 2 | 65200938 | 0 | within | 2.46E-07 |
| P-LCR | ss131564910 | 2 | 64970665 | CLEC17A | 92338 | 3.71E-06 |
| P-LCR | ss131056081 | 2 | 65057680 | CLEC17A | 5323 | 3.71E-06 |
| P-LCR | ss131191392 | 2 | 54474152 | JMJD4 | 54026 | 5.20E-06 |
| P-LCR | ss478944677 | 2 | 55226096 | SH3BP5L | within | 7.39E-06 |
| P-LCR | ss131085967 | 2 | 55240133 | SH3BP5L | 2589 | 8.02E-06 |
| P-LCR | ss478937495 | 2 | 70246409 | SMARCA4 | within | 1.04E-05 |
| P-LCR | ss131317336 | 6 | 157470018 | NFYC | within | 1.53E-05 |
| P-LCR | ss131189769 | 2 | 70506577 | ZNF653 | within | 1.77E-05 |
| P-LCR | ss131250184 | 4 | 141117946 | LMO4 | 223763 | 2.02E-05 |
| RBC | ss131276048 | 4 | 104825883 | INTS3 | within | 3.78E-06 |
| RBC | ss131493776 | 13 | 11696883 | UBE2E1 | within | 4.28E-06 |
| RBC | ss131206352 | 2 | 146082066 | KDM3B | within | 9.94E-06 |
| RBC | ss131276192 | 4 | 105017514 | PGLYRP4 | 26102 | 2.24E-05 |
| RDW | ss107831331 | 2 | 70061497 | CARM1 | within | 2.10E-07 |
| RDW | ss478944677 | 2 | 55226096 | SH3BP5L | within | 1.86E-06 |
| RDW | ss131085967 | 2 | 55240133 | SH3BP5L | 2589 | 2.05E-06 |
| RDW | ss131190995 | 2 | 59957369 | HAUS8 | within | 2.39E-06 |
| RDW | ss131190939 | 2 | 60261433 | CPAMD8 | within | 3.70E-06 |
| RDW | ss107884309 | 2 | 53681830 | 0 | 8852 | 6.33E-06 |
| RDW | ss107875173 | 2 | 161857853 | 0 | 10416 | 8.25E-06 |
| RDW | ss131190969 | 2 | 60202374 | CPAMD8 | within | 8.59E-06 |
| RDW | ss131541937 | 16 | 12945435 | U6 | 268 | 1.25E-05 |
| RDW | ss131189234 | 2 | 51376792 | GALNTL4 | within | 1.27E-05 |
| RDW | ss131033234 | 2 | 55304153 | 0 | 42870 | 1.36E-05 |
| RDW | ss478937548 | 2 | 89593299 | 0 | within | 1.38E-05 |
| RDW | ss478937928 | 2 | 90176527 | PAPD4 | within | 1.53E-05 |
| WBC | ss107857076 | 2 | 105499649 | 0 | 95033 | 6.03E-06 |
| WBC | ss131195511 | 2 | 101149437 | GPR98 | 277118 | 7.58E-06 |

The associated interval is defined as the region in which the distance between any two neighboring genome-wide significant SNPs is less than 10 Mb.

^1^The abbreviations of hematological traits are given in Table1. e.g. MCV is Mean corpuscular volume.

^2,3^ Chromosomal locations and positions of all significant SNP associated with hematological traits in Sus scrofa Build 10.2 assembly.

^4^Annotated gene which is nearest to the significant SNPs. The annotated gene database is from http://asia.ensembl.org/index.html.

^5^SNP designated as in a gene or distance (bp) from a gene region in Sus scrofa Build 10.2 assembly; “0” in column 6.represent un-annotated genes.
