# Supplementary figures and images for: Genome-wide association studies for hematological traits in Chinese Sutai pigs
Source: BMC Genet. 2014 Mar 27;15:41. doi: 10.1186/1471-2156-15-41 (PMC3986688; doi:10.1186/1471-2156-15-41)

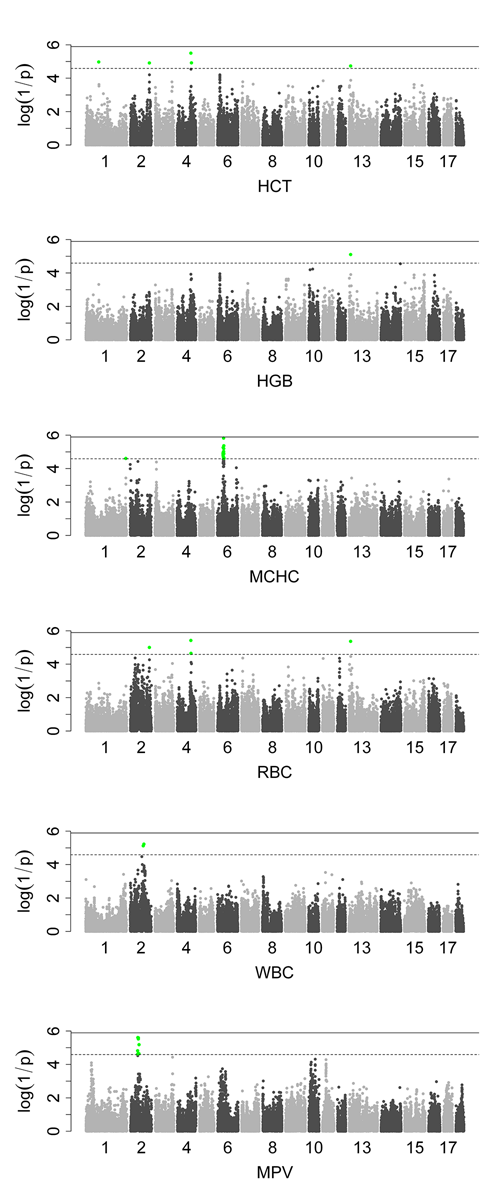

Supplement: Additional file 2: Figure S1 — Manhattan plots for the single marker analysis of hematological traits surpass suggestive significant threshold. log10 (1/P-value) values are shown for all SNPs that passed quality control. The solid line and dotted line denotes the Bonferroni-corrected genome-wide and suggestive significant threshold respectively. SNPs reaching the suggestive threshold are highlighted in green. HCT: hematocrit; HGB: hemoglobin; MCHC: mean corpuscular hemoglobin content; RBC: red blood cell; WBC: white blood cell count; MPV: mean platelet volume. [file 1471-2156-15-41-S2.tiff]

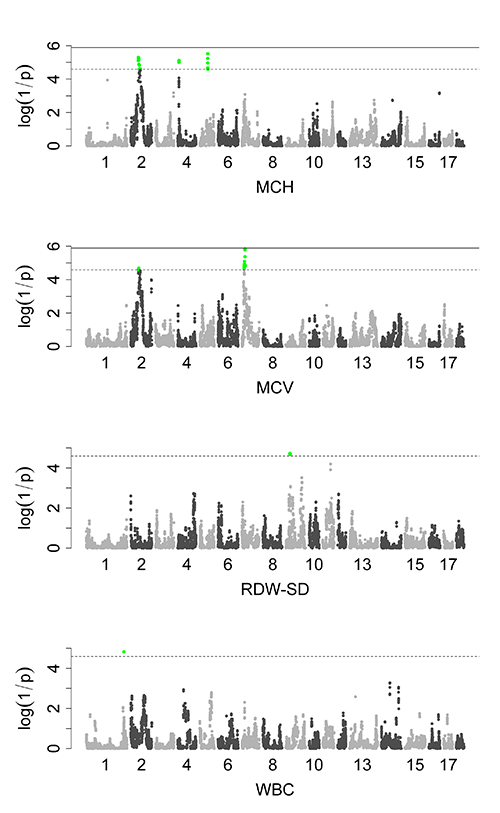

Supplement: Additional file 4: Figure S2 — Manhattan plots for the haplotype analysis of hematological traits surpass suggestive significant threshold. log10 (1/P-value) values are shown for all SNPs that passed quality control. The solid line and dotted line denotes the Bonferroni-corrected genome-wide and suggestive significant threshold respectively. SNPs reaching the suggestive threshold are highlighted in green. MCH: mean corpuscular hemoglobin; MCV: mean corpuscular volume; RDW-SD: red blood cell volume distribution width-SD; WBC: white blood cell count. [file 1471-2156-15-41-S4.tiff]
