# Supplementary material for: Genome-wide association studies for hematological traits in Chinese Sutai pigs
Source: BMC Genet. 2014 Mar 27;15:41. doi: 10.1186/1471-2156-15-41 (PMC3986688; doi:10.1186/1471-2156-15-41)
Supplement: Additional file 3: Table S2 — Description of all identified SNPs showing significant association with hematological traits by haplotype analysis. [file 1471-2156-15-41-S3.docx]

**Table S2 Description of all identified SNPs showing significant association with hematological traits by haplotype analysis**

| Traits^1^ | SNP name | Chr^2^ | Pos(bp)^3^ | Nearest gene^4^ | Distance（bp)^5^ | P-value |
| --- | --- | --- | --- | --- | --- | --- |
| HCT | ss131161664 | 1 | 161480924 | NFATC1 | 46745 | 1.15E-06 |
| HCT | ss107820868 | 1 | 161584404 | NFATC1 | within | 1.15E-06 |
| HCT | ss131044918 | 1 | 161993748 | SALL3 | 139806 | 1.15E-06 |
| HCT | ss131048937 | 1 | 162006116 | SALL3 | 152174 | 1.71E-06 |
| HCT | ss107855399 | 1 | 162026018 | SALL3 | 172076 | 1.62E-06 |
| HCT | ss107865395 | 1 | 162064446 | SALL3 | 210504 | 9.27E-06 |
| HCT | ss131338633 | 7 | 22852252 | 0 | 6993 | 1.89E-07 |
| HCT | ss131338696 | 7 | 22987329 | 0 | 42750 | 1.89E-07 |
| HCT | ss131338723 | 7 | 23022559 | 0 | 7520 | 1.89E-07 |
| HCT | ss131338784 | 7 | 23184219 | 0 | 2453 | 1.89E-07 |
| HCT | ss107810103 | 7 | 23206164 | OR2B2 | within | 1.89E-07 |
| HCT | ss131338806 | 7 | 23229208 | 0 | 2291 | 1.89E-07 |
| HCT | ss131042551 | 7 | 23706793 | 0 | within | 1.61E-07 |
| HCT | ss131338867 | 7 | 23878556 | ZNF165 | 545 | 1.31E-07 |
| HCT | ss131338914 | 7 | 23946632 | 0 | within | 1.40E-08 |
| HCT | ss131338934 | 7 | 23977356 | 0 | within | 1.40E-08 |
| HCT | ss131338961 | 7 | 24017747 | ZNF193 | 8228 | 1.40E-08 |
| HCT | ss131338967 | 7 | 24026461 | ZNF193 | within | 1.40E-08 |
| HCT | ss131339002 | 7 | 24070676 | 0 | within | 1.40E-08 |
| HCT | ss131339014 | 7 | 24096043 | PGBD1 | within | 1.40E-08 |
| HCT | ss131339018 | 7 | 24116868 | ZNF323 | 571 | 1.40E-08 |
| HCT | ss131339029 | 7 | 24128889 | ZSCAN12 | 3200 | 1.40E-08 |
| HCT | ss131339034 | 7 | 24149811 | ZSCAN12 | within | 1.40E-08 |
| HCT | ss107838291 | 7 | 24223914 | GPX5 | 9606 | 1.40E-08 |
| HCT | ss107865868 | 7 | 24320440 | 0 | 21614 | 1.40E-08 |
| HCT | ss131339140 | 7 | 24451815 | TRIM27 | 202 | 1.40E-08 |
| HCT | ss131339160 | 7 | 24480246 | Metazoa_SRP | 3914 | 1.40E-08 |
| HCT | ss107897931 | 7 | 24540590 | 0 | 1269 | 1.40E-08 |
| HCT | ss107842725 | 7 | 24777963 | CH242-196B23.2 | within | 1.28E-08 |
| HCT | ss131107260 | 8 | 22541053 | 0 | 251944 | 1.18E-06 |
| HCT | ss131384300 | 8 | 22676956 | 0 | 116041 | 2.04E-07 |
| HCT | ss131384323 | 8 | 22729045 | 0 | 63952 | 2.04E-07 |
| HCT | ss131384263 | 8 | 22805463 | 0 | 11477 | 2.21E-07 |
| HCT | ss131042035 | 8 | 22863583 | 0 | 69597 | 2.21E-07 |
| HCT | ss107865354 | 8 | 22904022 | 0 | 110036 | 2.21E-07 |
| HCT | ss478935853 | 8 | 23033602 | 0 | 239616 | 1.29E-07 |
| HCT | ss131384351 | 8 | 23147683 | 0 | 353697 | 2.05E-07 |
| HCT | ss131384346 | 8 | 23161471 | 0 | 367485 | 2.05E-07 |
| HCT | ss131384364 | 8 | 23234622 | 0 | 440636 | 2.05E-07 |
| HCT | ss131384383 | 8 | 23269849 | 0 | 475863 | 2.05E-07 |
| HCT | ss131389312 | 9 | 4942488 | 0 | within | 5.74E-06 |
| HCT | ss131389548 | 9 | 4990218 | 0 | 1140 | 9.73E-06 |
| HCT | ss131389950 | 9 | 5159477 | 0 | 7518 | 1.25E-05 |
| HCT | ss131392146 | 9 | 5669150 | OR51B2 | 15428 | 1.27E-05 |
| HCT | ss131392674 | 9 | 5815331 | OR51L1 | 1486 | 1.30E-05 |
| HCT | ss131392820 | 9 | 5851208 | OR51G2 | 834 | 1.30E-05 |
| HCT | ss107859199 | 9 | 5886633 | OR51T1 | 5983 | 1.30E-05 |
| HCT | ss131393045 | 9 | 5906685 | OR51H1P | 4536 | 9.84E-07 |
| HCT | ss131393315 | 9 | 5961665 | OR52R1 | 1478 | 9.60E-07 |
| HCT | ss120026798 | 9 | 5988229 | 0 | 2666 | 9.60E-07 |
| HCT | ss131064069 | 9 | 6061399 | OR51C1P | 2704 | 9.67E-07 |
| HCT | ss131567535 | 9 | 6098519 | OR51E1 | 10321 | 4.54E-07 |
| HCT | ss131393866 | 9 | 6152119 | 0 | 2563 | 4.54E-07 |
| HCT | ss131567781 | 9 | 6217480 | OR52K1 | 26136 | 4.54E-07 |
| HCT | ss131567775 | 9 | 6244800 | OR52K1 | within | 4.54E-07 |
| HCT | ss131122406 | 9 | 6250662 | OR52K1 | 5212 | 4.54E-07 |
| HCT | ss131062060 | 9 | 6287759 | 0 | 3582 | 4.54E-07 |
| HCT | ss131124047 | 9 | 6308875 | 0 | 20291 | 4.54E-07 |
| HCT | ss131097570 | 9 | 6476851 | OR52K2 | 11484 | 4.67E-07 |
| HCT | ss131102707 | 9 | 6480588 | OR52K2 | 7747 | 4.67E-07 |
| HCT | ss131393998 | 9 | 6491984 | OR52K2 | 2101 | 4.67E-07 |
| HCT | ss131394304 | 9 | 6538230 | TRIM21 | within | 4.67E-07 |
| HCT | ss131394390 | 9 | 6612868 | 0 | 9928 | 4.67E-07 |
| HCT | ss131395367 | 9 | 6928041 | STIM1 | within | 4.32E-07 |
| HCT | ss131395598 | 9 | 6964469 | STIM1 | within | 6.67E-07 |
| HCT | ss131395907 | 9 | 7010644 | STIM1 | within | 7.41E-07 |
| HCT | ss131396649 | 9 | 7041512 | STIM1 | within | 7.41E-07 |
| HCT | ss131396332 | 9 | 7069360 | RHOG | 11586 | 7.41E-07 |
| HCT | ss131396169 | 9 | 7086631 | RHOG | 5321 | 7.41E-07 |
| HCT | ss131396484 | 9 | 7123289 | NUP98 | within | 6.53E-07 |
| HCT | ss131396288 | 9 | 7149956 | NUP98 | within | 5.49E-07 |
| HCT | ss131076101 | 9 | 7178792 | NUP98 | within | 5.49E-07 |
| HCT | ss131055147 | 9 | 7211004 | ART5 | within | 4.09E-07 |
| HCT | ss131082449 | 9 | 7238788 | 0 | within | 4.09E-07 |
| HCT | ss131041658 | 9 | 7245993 | 0 | within | 4.09E-07 |
| HCT | ss478935847 | 9 | 7273496 | RNF121 | 20022 | 4.09E-07 |
| HCT | ss131397050 | 9 | 7334239 | RNF121 | within | 4.52E-07 |
| HCT | ss131396978 | 9 | 7350509 | IL18BP | 239 | 4.52E-07 |
| HCT | ss131396858 | 9 | 7371882 | NUMA1 | within | 4.52E-07 |
| HCT | ss131396818 | 9 | 7392224 | NUMA1 | 9789 | 4.52E-07 |
| HCT | ss107879930 | 9 | 7403995 | LRTOMT | 20631 | 4.52E-07 |
| HCT | ss131396789 | 9 | 7429068 | LRTOMT | within | 4.52E-07 |
| HCT | ss131086422 | 9 | 7449894 | ANAPC15 | within | 4.52E-07 |
| HCT | ss107807250 | 9 | 7469352 | 0 | 11615 | 4.52E-07 |
| HCT | ss131095060 | 9 | 7480997 | 0 | within | 4.52E-07 |
| HCT | ss131079823 | 9 | 7534515 | U6 | 6499 | 4.52E-07 |
| HCT | ss131096521 | 9 | 7550070 | FOLR2 | within | 7.23E-07 |
| HCT | ss131077503 | 9 | 7552010 | FOLR2 | 62 | 7.23E-07 |
| HCT | ss131032494 | 9 | 7570272 | INPPL1 | within | 7.23E-07 |
| HCT | ss131045638 | 9 | 7629459 | CLPB | within | 8.19E-07 |
| HCT | ss131127436 | 9 | 7629819 | CLPB | within | 8.19E-07 |
| HCT | ss131048361 | 9 | 7666097 | CLPB | 6551 | 8.19E-07 |
| HCT | ss131089553 | 9 | 7755887 | PDE2A | 5655 | 7.60E-07 |
| HCT | ss131066415 | 9 | 7758554 | PDE2A | 8322 | 8.68E-07 |
| HCT | ss131569383 | 9 | 7926117 | PDE2A | within | 5.31E-07 |
| HCT | ss131567760 | 9 | 7928669 | PDE2A | 131 | 5.31E-07 |
| HCT | ss131397383 | 9 | 8047973 | ARAP1 | 9232 | 5.14E-07 |
| HCT | ss478942422 | 11 | 60224450 | SLITRK1 | 55614 | 1.71E-06 |
| HCT | ss131445348 | 11 | 60266025 | SLITRK1 | 97189 | 1.71E-06 |
| HCT | ss107905315 | 12 | 15883731 | 41343 | 13950 | 1.10E-06 |
| HCT | ss107814716 | 12 | 15896241 | 41343 | 1440 | 9.63E-07 |
| HCT | ss131475641 | 12 | 15923014 | 41343 | within | 9.63E-07 |
| HCT | ss131475689 | 12 | 15953277 | MRC2 | within | 9.63E-07 |
| HCT | ss131475725 | 12 | 15974791 | MRC2 | within | 9.63E-07 |
| HCT | ss107797335 | 12 | 16041029 | TLK2 | within | 9.98E-07 |
| HCT | ss107904642 | 12 | 16142570 | EFCAB3 | 8757 | 9.98E-07 |
| HCT | ss107882981 | 12 | 16200087 | METTL2A | within | 1.93E-06 |
| HCT | ss131475805 | 12 | 16218533 | METTL2A | 17754 | 1.93E-06 |
| HCT | ss131091700 | 12 | 16298385 | METTL2A | 97606 | 1.93E-06 |
| HCT | ss131062928 | 12 | 16548593 | C17ORF57 | 72564 | 1.76E-06 |
| HCT | ss107831208 | 12 | 16579633 | C17ORF57 | 41524 | 1.78E-06 |
| HCT | ss131475842 | 12 | 16590416 | C17ORF57 | 30741 | 1.78E-06 |
| HCT | ss120027463 | 12 | 16627750 | C17ORF57 | within | 1.78E-06 |
| HCT | ss131475863 | 12 | 16671630 | C17ORF57 | within | 1.75E-06 |
| HCT | ss131051495 | 12 | 16702754 | C17ORF57 | within | 1.75E-06 |
| HCT | ss131104830 | 12 | 16710957 | CD61 | 6797 | 1.75E-06 |
| HCT | ss131475869 | 12 | 16721302 | CD61 | within | 1.75E-06 |
| HCT | ss131475896 | 12 | 16757288 | CD61 | within | 1.75E-06 |
| HCT | ss131475939 | 12 | 16784171 | CD61 | 8702 | 1.75E-06 |
| HCT | ss131475957 | 12 | 16800353 | MYL4 | within | 1.75E-06 |
| HCT | ss131475969 | 12 | 16816869 | MYL4 | 3750 | 1.75E-06 |
| HCT | ss131068409 | 12 | 16922755 | KIAA1267 | 2404 | 1.75E-06 |
| HCT | ss131476145 | 12 | 17081939 | KIAA1267 | within | 1.75E-06 |
| HCT | ss131476173 | 12 | 17124761 | MAPT | within | 1.56E-06 |
| HCT | ss131476224 | 12 | 17158049 | MAPT | within | 1.56E-06 |
| HCT | ss131119292 | 12 | 17271348 | 0 | 27056 | 1.73E-06 |
| HCT | ss131069074 | 12 | 17311437 | SPPL2C | 43435 | 1.73E-06 |
| HCT | ss131476021 | 12 | 17351240 | SPPL2C | 3632 | 1.73E-06 |
| HCT | ss131476012 | 12 | 17364341 | CRHR1 | 1802 | 1.73E-06 |
| HCT | ss131091421 | 12 | 17403368 | CRHR1 | within | 1.64E-06 |
| HCT | ss107860142 | 12 | 17487606 | ARL17A | 7900 | 1.25E-06 |
| HCT | ss131498830 | 14 | 116602344 | SORBS1 | within | 7.84E-07 |
| HCT | ss131498867 | 14 | 116650528 | SORBS1 | 2801 | 7.84E-07 |
| HCT | ss131498871 | 14 | 116664345 | SORBS1 | 16618 | 7.84E-07 |
| HCT | ss131498882 | 14 | 116688420 | SORBS1 | 40693 | 7.84E-07 |
| HCT | ss131498898 | 14 | 116719950 | SORBS1 | 72223 | 8.50E-07 |
| HCT | ss131498909 | 14 | 116740764 | ALDH18A1 | 67155 | 8.50E-07 |
| HCT | ss131498912 | 14 | 116759572 | ALDH18A1 | 48347 | 8.50E-07 |
| HCT | ss131498915 | 14 | 116773141 | ALDH18A1 | 34778 | 8.50E-07 |
| HCT | ss131498917 | 14 | 116796765 | ALDH18A1 | 11154 | 8.50E-07 |
| HCT | ss478936728 | 14 | 116827002 | ALDH18A1 | within | 8.50E-07 |
| HCT | ss131498923 | 14 | 116855028 | ALDH18A1 | within | 8.50E-07 |
| HCT | ss107858903 | 14 | 116890095 | TCTN3 | within | 8.50E-07 |
| HCT | ss131498932 | 14 | 116932140 | ENTPD1 | within | 8.50E-07 |
| HCT | ss107905276 | 14 | 116970199 | ENTPD1 | within | 8.50E-07 |
| HCT | ss131498942 | 14 | 117011153 | ENTPD1 | within | 8.50E-07 |
| HCT | ss120027907 | 14 | 117091767 | 0 | 1977 | 8.50E-07 |
| HCT | ss131498947 | 14 | 117112320 | 0 | 600 | 8.50E-07 |
| HCT | ss120028056 | 14 | 117140679 | 0 | 2303 | 8.50E-07 |
| HCT | ss107796442 | 14 | 117218440 | ZNF518B | 28256 | 8.50E-07 |
| HCT | ss120028520 | 14 | 117231943 | ZNF518B | 14753 | 8.50E-07 |
| HCT | ss131498966 | 14 | 117255863 | ZNF518B | 4692 | 8.50E-07 |
| HCT | ss131498969 | 14 | 117281061 | BLNK | within | 8.50E-07 |
| HCT | ss131498979 | 14 | 117312389 | BLNK | within | 8.50E-07 |
| HCT | ss131498984 | 14 | 117360297 | BLNK | 10332 | 8.31E-07 |
| HCT | ss131498990 | 14 | 117381848 | DNTT | 3607 | 8.31E-07 |
| HCT | ss131499002 | 14 | 117406540 | DNTT | within | 8.31E-07 |
| HCT | ss131499018 | 14 | 117428731 | OPALIN | within | 8.31E-07 |
| HCT | ss131499036 | 14 | 117485251 | TLL2 | within | 8.31E-07 |
| HCT | ss131499053 | 14 | 117506320 | TLL2 | within | 8.31E-07 |
| HCT | ss131499069 | 14 | 117562255 | TM9SF3 | 40357 | 8.31E-07 |
| HCT | ss131499085 | 14 | 117585705 | TM9SF3 | 16907 | 8.36E-07 |
| HCT | ss478943144 | 14 | 117647338 | TM9SF3 | within | 6.70E-07 |
| HCT | ss478943146 | 14 | 117666619 | TM9SF3 | within | 6.70E-07 |
| HCT | ss131499093 | 14 | 117685421 | PIK3AP1 | within | 6.70E-07 |
| HCT | ss131499103 | 14 | 117715367 | PIK3AP1 | within | 6.70E-07 |
| HCT | ss478943148 | 14 | 117752978 | PIK3AP1 | within | 6.70E-07 |
| HCT | ss131499118 | 14 | 117772183 | PIK3AP1 | within | 6.70E-07 |
| HCT | ss131499129 | 14 | 117821974 | U6 | 9924 | 5.93E-07 |
| HCT | ss131499134 | 14 | 117846180 | U6 | 34130 | 5.93E-07 |
| HCT | ss131499141 | 14 | 117886869 | U6 | 74819 | 5.93E-07 |
| HCT | ss131499160 | 14 | 118017826 | LCOR | 19295 | 5.93E-07 |
| HCT | ss478936597 | 14 | 118040644 | LCOR | within | 5.93E-07 |
| HCT | ss131499171 | 14 | 118072947 | C10ORF12 | 4197 | 5.93E-07 |
| HCT | ss131499201 | 14 | 118099509 | SLIT1 | within | 5.93E-07 |
| HCT | ss478935574 | 15 | 2729859 | KIF5C | 500 | 5.88E-06 |
| HCT | ss107871474 | 15 | 2744312 | KIF5C | within | 5.88E-06 |
| HCT | ss131075644 | 15 | 2746131 | KIF5C | within | 5.88E-06 |
| HCT | ss131062007 | 15 | 2748414 | KIF5C | within | 5.88E-06 |
| HCT | ss131569346 | 15 | 2788023 | KIF5C | within | 6.09E-06 |
| HCT | ss131121102 | 15 | 2798633 | KIF5C | within | 6.09E-06 |
| HCT | ss478939165 | 15 | 2841450 | KIF5C | within | 6.09E-06 |
| HCT | ss131093790 | 15 | 2844022 | KIF5C | within | 6.09E-06 |
| HCT | ss131525580 | 15 | 2916827 | KIF5C | 15262 | 6.40E-06 |
| HCT | ss131079813 | 15 | 2955185 | EPC2 | 25235 | 6.40E-06 |
| HCT | ss107829095 | 15 | 2964759 | EPC2 | 15661 | 6.40E-06 |
| HCT | ss131061216 | 15 | 2977314 | EPC2 | 3106 | 6.40E-06 |
| HCT | ss107800495 | 15 | 2990508 | EPC2 | within | 6.40E-06 |
| HCT | ss131084576 | 15 | 3017242 | EPC2 | within | 6.40E-06 |
| HCT | ss131524095 | 15 | 3082864 | EPC2 | within | 1.23E-05 |
| HCT | ss131527025 | 15 | 3145667 | EPC2 | 24209 | 1.23E-05 |
| HCT | ss131522031 | 15 | 3176420 | EPC2 | 54962 | 1.08E-05 |
| MCH | ss131191208 | 2 | 58824238 | COMP | 863 | 7.84E-06 |
| MCH | ss131191220 | 2 | 58871412 | CRTC1 | within | 7.84E-06 |
| MCH | ss478940495 | 2 | 59306764 | IL12RB1 | 2023 | 5.15E-06 |
| MCH | ss16337485 | 2 | 59391108 | CCDC124 | within | 5.15E-06 |
| MCH | ss478938204 | 2 | 59447736 | NIS | within | 5.15E-06 |
| MCH | ss478940493 | 2 | 59489740 | JAK3 | within | 5.15E-06 |
| MCH | ss131062784 | 2 | 59597177 | MAP1S | within | 5.15E-06 |
| MCH | ss131191098 | 2 | 59741934 | FAM129C | within | 4.98E-06 |
| MCH | ss131191069 | 2 | 59777365 | PGLS | 1279 | 4.98E-06 |
| MCH | ss131191075 | 2 | 59867578 | NXNL1 | within | 6.27E-06 |
| MCH | ss131190995 | 2 | 59957369 | HAUS8 | within | 6.27E-06 |
| MCH | ss131190993 | 2 | 60022633 | 0 | within | 6.27E-06 |
| MCH | ss131190984 | 2 | 60056520 | 0 | within | 6.27E-06 |
| MCH | ss131191047 | 2 | 60104724 | NR2F6 | within | 6.27E-06 |
| MCH | ss131190969 | 2 | 60202374 | CPAMD8 | within | 7.62E-06 |
| MCH | ss131190955 | 2 | 60227081 | CPAMD8 | within | 7.62E-06 |
| MCH | ss131190939 | 2 | 60261433 | CPAMD8 | within | 7.62E-06 |
| MCH | ss478936219 | 2 | 60289565 | CPAMD8 | within | 7.36E-06 |
| MCH | ss131190887 | 2 | 60318677 | SIN3B | within | 7.36E-06 |
| MCH | ss131190872 | 2 | 60338816 | SIN3B | within | 7.36E-06 |
| MCH | ss120020070 | 2 | 60773602 | CALR3 | 6340 | 7.36E-06 |
| MCH | ss131190801 | 2 | 60907615 | 0 | 1632 | 7.36E-06 |
| MCH | ss107880661 | 2 | 61592366 | CYP4F22 | 11496 | 5.80E-06 |
| MCH | ss131190713 | 2 | 61822605 | BRD4 | 5277 | 5.80E-06 |
| MCH | ss131190707 | 2 | 62081053 | CASP14 | 1478 | 6.07E-06 |
| MCH | ss131190685 | 2 | 62146679 | CCDC105 | 5537 | 1.31E-05 |
| MCH | ss131190234 | 2 | 66048253 | CACNA1A | within | 1.44E-05 |
| MCH | ss131190233 | 2 | 66106026 | CACNA1A | within | 1.44E-05 |
| MCH | ss131190216 | 2 | 66118786 | CACNA1A | within | 1.44E-05 |
| MCH | ss131190211 | 2 | 66138044 | CACNA1A | within | 1.44E-05 |
| MCH | ss478936141 | 2 | 66167179 | CACNA1A | within | 1.44E-05 |
| MCH | ss131190204 | 2 | 66249504 | NACC1 | within | 1.44E-05 |
| MCH | ss131190194 | 2 | 66274569 | FARSA | within | 1.44E-05 |
| MCH | ss478940489 | 2 | 66374171 | NFIX | within | 1.50E-05 |
| MCH | ss131190148 | 2 | 66489142 | HOOK2 | within | 1.50E-05 |
| MCH | ss131190127 | 2 | 66651641 | TNPO2 | 468 | 1.50E-05 |
| MCH | ss131272108 | 4 | 10390156 | 0 | 271636 | 9.96E-06 |
| MCH | ss131272197 | 4 | 10404548 | 0 | 257244 | 9.96E-06 |
| MCH | ss131272330 | 4 | 10423196 | 0 | 238596 | 9.96E-06 |
| MCH | ss131272377 | 4 | 10447026 | 0 | 214766 | 9.96E-06 |
| MCH | ss131272422 | 4 | 10527117 | 0 | 134675 | 7.68E-06 |
| MCH | ss107879652 | 4 | 10565469 | 0 | 96323 | 7.68E-06 |
| MCH | ss131272693 | 4 | 10597928 | 0 | 63864 | 7.68E-06 |
| MCH | ss131272951 | 4 | 10645620 | 0 | 16172 | 8.14E-06 |
| MCH | ss131273877 | 4 | 10686323 | 0 | 17458 | 8.14E-06 |
| MCH | ss131273980 | 4 | 10703277 | 0 | 34412 | 8.14E-06 |
| MCH | ss131274033 | 4 | 10709751 | 0 | 40886 | 8.14E-06 |
| MCH | ss120030788 | 4 | 10740075 | FAM49B | 60991 | 8.14E-06 |
| MCH | ss131274293 | 4 | 10763544 | FAM49B | 37522 | 8.63E-06 |
| MCH | ss131274453 | 4 | 10815136 | FAM49B | within | 8.63E-06 |
| MCH | ss107835907 | 4 | 11002675 | 0 | 92377 | 8.57E-06 |
| MCH | ss107852849 | 5 | 61354749 | 0 | within | 5.76E-06 |
| MCH | ss131287311 | 5 | 61370123 | 0 | within | 5.76E-06 |
| MCH | ss107897662 | 5 | 61751304 | ATF7IP | 179819 | 1.08E-05 |
| MCH | ss131287363 | 5 | 61894232 | GRIN2B | 176139 | 3.05E-06 |
| MCH | ss131287380 | 5 | 61906403 | GRIN2B | 163968 | 3.05E-06 |
| MCH | ss478941323 | 5 | 61925570 | GRIN2B | 144801 | 3.05E-06 |
| MCH | ss131287386 | 5 | 61941547 | GRIN2B | 128824 | 2.17E-05 |
| MCH | ss131287393 | 5 | 61966057 | GRIN2B | 104314 | 2.13E-05 |
| MCH | ss131287397 | 5 | 61979581 | GRIN2B | 90790 | 2.13E-05 |
| MCV | ss131190707 | 2 | 62081053 | CASP14 | 1478 | 2.03E-05 |
| MCV | ss131336686 | 7 | 18300586 | PRL | 111907 | 2.23E-05 |
| MCV | ss131336716 | 7 | 18401242 | PRL | 11251 | 1.66E-05 |
| MCV | ss131336720 | 7 | 18421696 | PRL | within | 1.66E-05 |
| MCV | ss120019000 | 7 | 18460249 | PRL | 35407 | 1.66E-05 |
| MCV | ss107907529 | 7 | 18528143 | PRL | 103301 | 1.66E-05 |
| MCV | ss107817036 | 7 | 18545282 | HDGFL1 | 111452 | 1.66E-05 |
| MCV | ss131336778 | 7 | 18568640 | HDGFL1 | 88094 | 1.89E-05 |
| MCV | ss131336808 | 7 | 18668395 | HDGFL1 | 10975 | 1.89E-05 |
| MCV | ss131336814 | 7 | 18691549 | U1 | 10550 | 1.89E-05 |
| MCV | ss478938637 | 7 | 18714004 | U1 | 11743 | 1.89E-05 |
| MCV | ss131336844 | 7 | 18766174 | U1 | 63913 | 1.63E-05 |
| MCV | ss131336868 | 7 | 18796045 | U1 | 93784 | 1.58E-05 |
| MCV | ss107798230 | 7 | 18810414 | U1 | 108153 | 1.58E-05 |
| MCV | ss131336874 | 7 | 18822579 | U1 | 120318 | 1.58E-05 |
| MCV | ss131336877 | 7 | 18847348 | U1 | 145087 | 1.58E-05 |
| MCV | ss131336884 | 7 | 18869465 | U1 | 167204 | 1.58E-05 |
| MCV | ss131336894 | 7 | 18892726 | U1 | 190465 | 1.58E-05 |
| MCV | ss107837640 | 7 | 18912470 | U1 | 210209 | 1.58E-05 |
| MCV | ss120019117 | 7 | 18949401 | U1 | 247140 | 1.58E-05 |
| MCV | ss131336918 | 7 | 18984509 | U1 | 282248 | 1.58E-05 |
| MCV | ss478935472 | 7 | 19004434 | U1 | 302173 | 1.58E-05 |
| MCV | ss120018957 | 7 | 19232432 | U1 | 530171 | 1.22E-05 |
| MCV | ss131337006 | 7 | 19255552 | U1 | 553291 | 1.22E-05 |
| MCV | ss131337075 | 7 | 19547639 | SNORA70 | 408913 | 1.86E-05 |
| MCV | ss131337064 | 7 | 19586998 | SNORA70 | 369554 | 1.86E-05 |
| MCV | ss131337050 | 7 | 19607859 | SNORA70 | 348693 | 1.86E-05 |
| MCV | ss120018820 | 7 | 19631660 | SNORA70 | 324892 | 1.86E-05 |
| MCV | ss131337129 | 7 | 20072507 | NRSN1 | 84399 | 1.64E-05 |
| MCV | ss131337133 | 7 | 20097822 | NRSN1 | 59084 | 1.64E-05 |
| MCV | ss120018291 | 7 | 20159272 | NRSN1 | within | 1.64E-05 |
| MCV | ss131337151 | 7 | 20189185 | DCDC2 | 8697 | 1.64E-05 |
| MCV | ss120018907 | 7 | 20244257 | DCDC2 | within | 8.17E-06 |
| MCV | ss131337704 | 7 | 20985785 | CMAH | 46504 | 1.19E-05 |
| MCV | ss131337721 | 7 | 21006494 | CMAH | 25795 | 1.19E-05 |
| MCV | ss131338914 | 7 | 23946632 | 0 | within | 1.49E-06 |
| MCV | ss131338934 | 7 | 23977356 | 0 | within | 1.49E-06 |
| MCV | ss131338961 | 7 | 24017747 | ZNF193 | 8228 | 1.49E-06 |
| MCV | ss131338967 | 7 | 24026461 | ZNF193 | within | 1.49E-06 |
| MCV | ss131339002 | 7 | 24070676 | 0 | within | 1.49E-06 |
| MCV | ss131339014 | 7 | 24096043 | PGBD1 | within | 1.49E-06 |
| MCV | ss131339018 | 7 | 24116868 | ZNF323 | 571 | 1.49E-06 |
| MCV | ss131339029 | 7 | 24128889 | ZSCAN12 | 3200 | 1.49E-06 |
| MCV | ss131339034 | 7 | 24149811 | ZSCAN12 | within | 1.49E-06 |
| MCV | ss107838291 | 7 | 24223914 | GPX5 | 9606 | 1.49E-06 |
| MCV | ss107865868 | 7 | 24320440 | 0 | 21614 | 1.49E-06 |
| MCV | ss131339140 | 7 | 24451815 | TRIM27 | 202 | 1.49E-06 |
| MCV | ss131339160 | 7 | 24480246 | Metazoa_SRP | 3914 | 1.49E-06 |
| MCV | ss107897931 | 7 | 24540590 | 0 | 1269 | 1.49E-06 |
| MCV | ss107842725 | 7 | 24777963 | CH242-196B23.2 | within | 1.47E-06 |
| MCV | ss131339382 | 7 | 24791155 | TRIM26 | 4679 | 1.70E-06 |
| MCV | ss131339411 | 7 | 24817635 | TRIM26 | within | 4.26E-06 |
| MCV | ss107833692 | 7 | 24848207 | TRIM10 | within | 4.26E-06 |
| MCV | ss478936712 | 7 | 26822610 | FB19 | within | 1.48E-05 |
| MCV | ss478936577 | 7 | 26954317 | MDC1 | within | 1.48E-05 |
| MCV | ss131340120 | 7 | 27019705 | SBAB-353A11.2 | 3162 | 1.48E-05 |
| MCV | ss131340150 | 7 | 27082547 | DDR | 13486 | 1.48E-05 |
| MCV | ss131340170 | 7 | 27103675 | DDR | within | 1.48E-05 |
| MCV | ss478936930 | 7 | 27128290 | VARS2 | within | 1.48E-05 |
| MCV | ss131340325 | 7 | 27300895 | SLA-8 | within | 1.48E-05 |
| MCV | ss131340303 | 7 | 27317882 | SLA-8 | within | 1.48E-05 |
| MCV | ss131340297 | 7 | 27329622 | SLA-8 | within | 1.48E-05 |
| MCV | ss131340257 | 7 | 27352011 | SLA-8 | within | 1.48E-05 |
| MCV | ss131340253 | 7 | 27373527 | SLA-8 | within | 1.48E-05 |
| MCV | ss131340243 | 7 | 27389510 | SLA-8 | within | 1.48E-05 |
| MCV | ss131340499 | 7 | 27470374 | SLA-8 | within | 1.42E-05 |
| MCV | ss23131766 | 7 | 27497439 | SLA-8 | within | 1.42E-05 |
| MCV | ss131340433 | 7 | 27619988 | SLA-8 | within | 1.42E-05 |
| RBC | ss131161664 | 1 | 161480924 | NFATC1 | 46745 | 2.83E-06 |
| RBC | ss107820868 | 1 | 161584404 | NFATC1 | within | 2.83E-06 |
| RBC | ss131044918 | 1 | 161993748 | SALL3 | 139806 | 2.83E-06 |
| RBC | ss131048937 | 1 | 162006116 | SALL3 | 152174 | 5.83E-06 |
| RBC | ss107855399 | 1 | 162026018 | SALL3 | 172076 | 5.74E-06 |
| RBC | ss131338633 | 7 | 22852252 | 0 | 6993 | 8.49E-06 |
| RBC | ss131338696 | 7 | 22987329 | 0 | 42750 | 8.49E-06 |
| RBC | ss131338723 | 7 | 23022559 | 0 | 7520 | 8.49E-06 |
| RBC | ss131338784 | 7 | 23184219 | 0 | 2453 | 8.49E-06 |
| RBC | ss107810103 | 7 | 23206164 | OR2B2 | within | 8.49E-06 |
| RBC | ss131338806 | 7 | 23229208 | 0 | 2291 | 8.49E-06 |
| RBC | ss131042551 | 7 | 23706793 | 0 | within | 7.74E-06 |
| RBC | ss131338867 | 7 | 23878556 | ZNF165 | 545 | 7.89E-06 |
| RBC | ss131338914 | 7 | 23946632 | 0 | within | 1.25E-07 |
| RBC | ss131338934 | 7 | 23977356 | 0 | within | 1.25E-07 |
| RBC | ss131338961 | 7 | 24017747 | ZNF193 | 8228 | 1.25E-07 |
| RBC | ss131338967 | 7 | 24026461 | ZNF193 | within | 1.25E-07 |
| RBC | ss131339002 | 7 | 24070676 | 0 | within | 1.25E-07 |
| RBC | ss131339014 | 7 | 24096043 | PGBD1 | within | 1.25E-07 |
| RBC | ss131339018 | 7 | 24116868 | ZNF323 | 571 | 1.25E-07 |
| RBC | ss131339029 | 7 | 24128889 | ZSCAN12 | 3200 | 1.25E-07 |
| RBC | ss131339034 | 7 | 24149811 | ZSCAN12 | within | 1.25E-07 |
| RBC | ss107838291 | 7 | 24223914 | GPX5 | 9606 | 1.25E-07 |
| RBC | ss107865868 | 7 | 24320440 | 0 | 21614 | 1.25E-07 |
| RBC | ss131339140 | 7 | 24451815 | TRIM27 | 202 | 1.25E-07 |
| RBC | ss131339160 | 7 | 24480246 | Metazoa_SRP | 3914 | 1.25E-07 |
| RBC | ss107897931 | 7 | 24540590 | 0 | 1269 | 1.25E-07 |
| RBC | ss107842725 | 7 | 24777963 | CH242-196B23.2 | within | 1.20E-07 |
| RBC | ss131107260 | 8 | 22541053 | 0 | 251944 | 3.83E-06 |
| RBC | ss131384300 | 8 | 22676956 | 0 | 116041 | 3.24E-06 |
| RBC | ss131384323 | 8 | 22729045 | 0 | 63952 | 3.24E-06 |
| RBC | ss131384263 | 8 | 22805463 | 0 | 11477 | 3.45E-06 |
| RBC | ss131042035 | 8 | 22863583 | 0 | 69597 | 3.45E-06 |
| RBC | ss107865354 | 8 | 22904022 | 0 | 110036 | 3.45E-06 |
| RBC | ss478935853 | 8 | 23033602 | 0 | 239616 | 1.62E-06 |
| RBC | ss131384351 | 8 | 23147683 | 0 | 353697 | 2.38E-06 |
| RBC | ss131384346 | 8 | 23161471 | 0 | 367485 | 2.38E-06 |
| RBC | ss131384364 | 8 | 23234622 | 0 | 440636 | 2.38E-06 |
| RBC | ss131384383 | 8 | 23269849 | 0 | 475863 | 2.38E-06 |
| RBC | ss131393045 | 9 | 5906685 | OR51H1P | 4536 | 6.05E-06 |
| RBC | ss131393315 | 9 | 5961665 | OR52R1 | 1478 | 6.05E-06 |
| RBC | ss120026798 | 9 | 5988229 | 0 | 2666 | 6.05E-06 |
| RBC | ss131064069 | 9 | 6061399 | OR51C1P | 2704 | 6.05E-06 |
| RBC | ss131567535 | 9 | 6098519 | OR51E1 | 10321 | 2.89E-06 |
| RBC | ss131393866 | 9 | 6152119 | 0 | 2563 | 2.89E-06 |
| RBC | ss131567781 | 9 | 6217480 | OR52K1 | 26136 | 2.89E-06 |
| RBC | ss131567775 | 9 | 6244800 | OR52K1 | within | 2.89E-06 |
| RBC | ss131122406 | 9 | 6250662 | OR52K1 | 5212 | 2.89E-06 |
| RBC | ss131062060 | 9 | 6287759 | 0 | 3582 | 2.89E-06 |
| RBC | ss131124047 | 9 | 6308875 | 0 | 20291 | 2.89E-06 |
| RBC | ss131097570 | 9 | 6476851 | OR52K2 | 11484 | 2.91E-06 |
| RBC | ss131102707 | 9 | 6480588 | OR52K2 | 7747 | 2.91E-06 |
| RBC | ss131393998 | 9 | 6491984 | OR52K2 | 2101 | 2.91E-06 |
| RBC | ss131394304 | 9 | 6538230 | TRIM21 | within | 2.91E-06 |
| RBC | ss131394390 | 9 | 6612868 | 0 | 9928 | 2.91E-06 |
| RBC | ss131395367 | 9 | 6928041 | STIM1 | within | 2.85E-06 |
| RBC | ss131395598 | 9 | 6964469 | STIM1 | within | 3.73E-06 |
| RBC | ss131395907 | 9 | 7010644 | STIM1 | within | 4.03E-06 |
| RBC | ss131396649 | 9 | 7041512 | STIM1 | within | 4.03E-06 |
| RBC | ss131396332 | 9 | 7069360 | RHOG | 11586 | 4.03E-06 |
| RBC | ss131396169 | 9 | 7086631 | RHOG | 5321 | 4.03E-06 |
| RBC | ss131396484 | 9 | 7123289 | NUP98 | within | 3.64E-06 |
| RBC | ss131396288 | 9 | 7149956 | NUP98 | within | 3.24E-06 |
| RBC | ss131076101 | 9 | 7178792 | NUP98 | within | 3.24E-06 |
| RBC | ss131055147 | 9 | 7211004 | ART5 | within | 2.62E-06 |
| RBC | ss131082449 | 9 | 7238788 | 0 | within | 2.62E-06 |
| RBC | ss131041658 | 9 | 7245993 | 0 | within | 2.62E-06 |
| RBC | ss478935847 | 9 | 7273496 | RNF121 | 20022 | 2.62E-06 |
| RBC | ss131397050 | 9 | 7334239 | RNF121 | within | 2.70E-06 |
| RBC | ss131396978 | 9 | 7350509 | IL18BP | 239 | 2.70E-06 |
| RBC | ss131396858 | 9 | 7371882 | NUMA1 | within | 2.70E-06 |
| RBC | ss131396818 | 9 | 7392224 | NUMA1 | 9789 | 2.70E-06 |
| RBC | ss107879930 | 9 | 7403995 | LRTOMT | 20631 | 2.70E-06 |
| RBC | ss131396789 | 9 | 7429068 | LRTOMT | within | 2.70E-06 |
| RBC | ss131086422 | 9 | 7449894 | ANAPC15 | within | 2.70E-06 |
| RBC | ss107807250 | 9 | 7469352 | 0 | 11615 | 2.70E-06 |
| RBC | ss131095060 | 9 | 7480997 | 0 | within | 2.70E-06 |
| RBC | ss131079823 | 9 | 7534515 | U6 | 6499 | 2.70E-06 |
| RBC | ss131096521 | 9 | 7550070 | FOLR2 | within | 3.27E-06 |
| RBC | ss131077503 | 9 | 7552010 | FOLR2 | 62 | 3.27E-06 |
| RBC | ss131032494 | 9 | 7570272 | INPPL1 | within | 3.27E-06 |
| RBC | ss131045638 | 9 | 7629459 | CLPB | within | 3.76E-06 |
| RBC | ss131127436 | 9 | 7629819 | CLPB | within | 3.76E-06 |
| RBC | ss131048361 | 9 | 7666097 | CLPB | 6551 | 3.76E-06 |
| RBC | ss131089553 | 9 | 7755887 | PDE2A | 5655 | 3.34E-06 |
| RBC | ss131066415 | 9 | 7758554 | PDE2A | 8322 | 3.21E-06 |
| RBC | ss131569383 | 9 | 7926117 | PDE2A | within | 1.51E-06 |
| RBC | ss131567760 | 9 | 7928669 | PDE2A | 131 | 1.51E-06 |
| RBC | ss131397383 | 9 | 8047973 | ARAP1 | 9232 | 1.50E-06 |
| RBC | ss107905315 | 12 | 15883731 | 41343 | 13950 | 1.66E-06 |
| RBC | ss107814716 | 12 | 15896241 | 41343 | 1440 | 1.45E-06 |
| RBC | ss131475641 | 12 | 15923014 | 41343 | within | 1.45E-06 |
| RBC | ss131475689 | 12 | 15953277 | MRC2 | within | 1.45E-06 |
| RBC | ss131475725 | 12 | 15974791 | MRC2 | within | 1.45E-06 |
| RBC | ss107797335 | 12 | 16041029 | TLK2 | within | 1.60E-06 |
| RBC | ss107904642 | 12 | 16142570 | EFCAB3 | 8757 | 1.60E-06 |
| RBC | ss107882981 | 12 | 16200087 | METTL2A | within | 3.03E-06 |
| RBC | ss131475805 | 12 | 16218533 | METTL2A | 17754 | 3.03E-06 |
| RBC | ss131091700 | 12 | 16298385 | METTL2A | 97606 | 3.03E-06 |
| RBC | ss131062928 | 12 | 16548593 | C17ORF57 | 72564 | 3.15E-06 |
| RBC | ss107831208 | 12 | 16579633 | C17ORF57 | 41524 | 2.47E-06 |
| RBC | ss131475842 | 12 | 16590416 | C17ORF57 | 30741 | 2.47E-06 |
| RBC | ss120027463 | 12 | 16627750 | C17ORF57 | within | 2.47E-06 |
| RBC | ss131475863 | 12 | 16671630 | C17ORF57 | within | 2.44E-06 |
| RBC | ss131051495 | 12 | 16702754 | C17ORF57 | within | 2.44E-06 |
| RBC | ss131104830 | 12 | 16710957 | CD61 | 6797 | 2.44E-06 |
| RBC | ss131475869 | 12 | 16721302 | CD61 | within | 2.44E-06 |
| RBC | ss131475896 | 12 | 16757288 | CD61 | within | 2.44E-06 |
| RBC | ss131475939 | 12 | 16784171 | CD61 | 8702 | 2.44E-06 |
| RBC | ss131475957 | 12 | 16800353 | MYL4 | within | 2.44E-06 |
| RBC | ss131475969 | 12 | 16816869 | MYL4 | 3750 | 2.44E-06 |
| RBC | ss131068409 | 12 | 16922755 | KIAA1267 | 2404 | 2.44E-06 |
| RBC | ss131476145 | 12 | 17081939 | KIAA1267 | within | 2.44E-06 |
| RBC | ss131476173 | 12 | 17124761 | MAPT | within | 2.80E-06 |
| RBC | ss131476224 | 12 | 17158049 | MAPT | within | 2.80E-06 |
| RBC | ss131119292 | 12 | 17271348 | 0 | 27056 | 2.81E-06 |
| RBC | ss131069074 | 12 | 17311437 | SPPL2C | 43435 | 2.81E-06 |
| RBC | ss131476021 | 12 | 17351240 | SPPL2C | 3632 | 2.81E-06 |
| RBC | ss131476012 | 12 | 17364341 | CRHR1 | 1802 | 2.81E-06 |
| RBC | ss131091421 | 12 | 17403368 | CRHR1 | within | 2.56E-06 |
| RBC | ss107860142 | 12 | 17487606 | ARL17A | 7900 | 1.92E-06 |
| RBC | ss131498830 | 14 | 116602344 | SORBS1 | within | 5.01E-06 |
| RBC | ss131498867 | 14 | 116650528 | SORBS1 | 2801 | 5.01E-06 |
| RBC | ss131498871 | 14 | 116664345 | SORBS1 | 16618 | 5.01E-06 |
| RBC | ss131498882 | 14 | 116688420 | SORBS1 | 40693 | 5.01E-06 |
| RBC | ss131498898 | 14 | 116719950 | SORBS1 | 72223 | 5.06E-06 |
| RBC | ss131498909 | 14 | 116740764 | ALDH18A1 | 67155 | 5.06E-06 |
| RBC | ss131498912 | 14 | 116759572 | ALDH18A1 | 48347 | 5.06E-06 |
| RBC | ss131498915 | 14 | 116773141 | ALDH18A1 | 34778 | 5.06E-06 |
| RBC | ss131498917 | 14 | 116796765 | ALDH18A1 | 11154 | 5.06E-06 |
| RBC | ss478936728 | 14 | 116827002 | ALDH18A1 | within | 5.06E-06 |
| RBC | ss131498923 | 14 | 116855028 | ALDH18A1 | within | 5.06E-06 |
| RBC | ss107858903 | 14 | 116890095 | TCTN3 | within | 5.06E-06 |
| RBC | ss131498932 | 14 | 116932140 | ENTPD1 | within | 5.06E-06 |
| RBC | ss107905276 | 14 | 116970199 | ENTPD1 | within | 5.06E-06 |
| RBC | ss131498942 | 14 | 117011153 | ENTPD1 | within | 5.06E-06 |
| RBC | ss120027907 | 14 | 117091767 | 0 | 1977 | 5.06E-06 |
| RBC | ss131498947 | 14 | 117112320 | 0 | 600 | 5.06E-06 |
| RBC | ss120028056 | 14 | 117140679 | 0 | 2303 | 5.06E-06 |
| RBC | ss107796442 | 14 | 117218440 | ZNF518B | 28256 | 5.06E-06 |
| RBC | ss120028520 | 14 | 117231943 | ZNF518B | 14753 | 5.06E-06 |
| RBC | ss131498966 | 14 | 117255863 | ZNF518B | 4692 | 5.06E-06 |
| RBC | ss131498969 | 14 | 117281061 | BLNK | within | 5.06E-06 |
| RBC | ss131498979 | 14 | 117312389 | BLNK | within | 5.06E-06 |
| RBC | ss131498984 | 14 | 117360297 | BLNK | 10332 | 5.01E-06 |
| RBC | ss131498990 | 14 | 117381848 | DNTT | 3607 | 5.01E-06 |
| RBC | ss131499002 | 14 | 117406540 | DNTT | within | 5.01E-06 |
| RBC | ss131499018 | 14 | 117428731 | OPALIN | within | 5.01E-06 |
| RBC | ss131499036 | 14 | 117485251 | TLL2 | within | 5.01E-06 |
| RBC | ss131499053 | 14 | 117506320 | TLL2 | within | 5.01E-06 |
| RBC | ss131499069 | 14 | 117562255 | TM9SF3 | 40357 | 5.01E-06 |
| RBC | ss131499085 | 14 | 117585705 | TM9SF3 | 16907 | 4.96E-06 |
| RBC | ss478943144 | 14 | 117647338 | TM9SF3 | within | 4.59E-06 |
| RBC | ss478943146 | 14 | 117666619 | TM9SF3 | within | 4.59E-06 |
| RBC | ss131499093 | 14 | 117685421 | PIK3AP1 | within | 4.59E-06 |
| RBC | ss131499103 | 14 | 117715367 | PIK3AP1 | within | 4.59E-06 |
| RBC | ss478943148 | 14 | 117752978 | PIK3AP1 | within | 4.59E-06 |
| RBC | ss131499118 | 14 | 117772183 | PIK3AP1 | within | 4.59E-06 |
| RBC | ss131499129 | 14 | 117821974 | U6 | 9924 | 4.36E-06 |
| RBC | ss131499134 | 14 | 117846180 | U6 | 34130 | 4.36E-06 |
| RBC | ss131499141 | 14 | 117886869 | U6 | 74819 | 4.36E-06 |
| RBC | ss131499160 | 14 | 118017826 | LCOR | 19295 | 4.36E-06 |
| RBC | ss478936597 | 14 | 118040644 | LCOR | within | 4.36E-06 |
| RBC | ss131499171 | 14 | 118072947 | C10ORF12 | 4197 | 4.36E-06 |
| RBC | ss131499201 | 14 | 118099509 | SLIT1 | within | 4.36E-06 |
| RDW-SD | ss107895209 | 9 | 33199173 | 0 | 129000 | 1.89E-05 |
| RDW-SD | ss131114130 | 9 | 33331814 | 0 | 3311 | 1.89E-05 |
| RDW-SD | ss131107191 | 9 | 33899905 | U6 | 483660 | 2.14E-05 |
| RDW-SD | ss107888504 | 9 | 34148025 | U6 | 731780 | 1.97E-05 |
| RDW-SD | ss120026884 | 9 | 34331512 | CNTN5 | 757213 | 1.97E-05 |
| RDW-SD | ss131388067 | 9 | 34372328 | CNTN5 | 716397 | 1.97E-05 |
| RDW-SD | ss478944331 | 9 | 34393310 | CNTN5 | 695415 | 1.97E-05 |
| RDW-SD | ss131388085 | 9 | 34531580 | CNTN5 | 557145 | 1.97E-05 |
| RDW-SD | ss131388088 | 9 | 34556085 | CNTN5 | 532640 | 1.97E-05 |
| RDW-SD | ss120026796 | 9 | 34624040 | CNTN5 | 464685 | 1.97E-05 |
| RDW-SD | ss131388120 | 9 | 34804811 | CNTN5 | 283914 | 1.97E-05 |
| RDW-SD | ss131388131 | 9 | 34841808 | CNTN5 | 246917 | 1.97E-05 |
| RDW-SD | ss107892416 | 9 | 34870189 | CNTN5 | 218536 | 1.97E-05 |
| WBC | ss131152863 | 1 | 289943447 | TLR4 | 157600 | 1.49E-05 |

The associated interval is defined as the region in which the distance between any two neighboring genome-wide significant SNPs is less than 10 Mb.

^1^The abbreviations of hematological traits are given in Table1. e.g. MCV is Mean corpuscular volume.

^2,3^ Chromosomal locations and positions of all significant SNP associated with hematological traits in Sus scrofa Build 10.2 assembly.

^4^Annotated gene which is nearest to the significant SNPs. The annotated gene database is from http://asia.ensembl.org/index.html.

^5^SNP designated as in a gene or distance (bp) from a gene region in Sus scrofa Build 10.2 assembly. “0” in column 6.represent un-annotated genes.
