# Supplementary material for: Genome-wide association studies for hematological traits in Chinese Sutai pigs
Source: BMC Genet. 2014 Mar 27;15:41. doi: 10.1186/1471-2156-15-41 (PMC3986688; doi:10.1186/1471-2156-15-41)
Supplement: Additional file 5: Table S3 — Description of correlation and P-value among the 15 hematological traits. [file 1471-2156-15-41-S5.docx]

**Table S3 Description of correlation and p-value among the 15 hematological traits**

|  | WBC | RBC | HGB | HCT | MCV | MCH | MCHC | RDW | PLT | MPV | PDW | LYM | LYMA | P-LCR | RDW-SD |
| --- | --- | --- | --- | --- | --- | --- | --- | --- | --- | --- | --- | --- | --- | --- | --- |
| WBC | 1 | 0.113 | -0.017 | 0.025 | -0.234 | -0.311 | -0.226 | 0.107 | 0.029 | -0.198 | -0.098 | 0.458 | -0.423 | -0.182 | -0.046 |
| RBC | 2.62E-02 | 1 | 0.844 | 0.924 | -0.314 | -0.542 | -0.495 | 0.534 | -0.192 | -0.137 | 0.017 | 0.002 | -0.123 | -0.194 | 0.119 |
| HGB | 7.47E-01 | < 1.0E-16 | 1 | 0.94 | 0.087 | -0.074 | -0.202 | 0.291 | -0.196 | 0.127 | 0.199 | -0.001 | -0.009 | 0.098 | 0.181 |
| HCT | 6.23E-01 | < 1.0E-16 | < 1.0E-16 | 1 | 0.067 | -0.251 | -0.481 | 0.404 | -0.230 | 0.086 | 0.189 | -0.023 | -0.058 | 0.049 | 0.241 |
| MCV | 3.65E-06 | 2.88E-10 | 8.81E-02 | 1.90E-01 | 1 | 0.804 | 0.082 | -0.382 | -0.071 | 0.551 | 0.414 | -0.050 | 0.189 | 0.586 | 0.300 |
| MCH | 5.16E-10 | < 1.0E-16 | 1.51E-01 | 6.23E-07 | < 1.0E-16 | 1 | 0.653 | -0.511 | 0.002 | 0.482 | 0.309 | -0.065 | 0.241 | 0.524 | 0.103 |
| MCHC | 8.43E-06 | < 1.0E-16 | 7.39E-05 | < 1.0E-16 | 1.08E-01 | < 1.0E-16 | 1 | -0.429 | 0.100 | 0.062 | -0.064 | -0.054 | 0.152 | 0.082 | -0.252 |
| RDW | 5.78E-02 | < 1.0E-16 | 1.51E-07 | 6.84E-14 | 1.88E-12 | < 1.0E-16 | 1.55E-15 | 1 | -0.082 | -0.153 | -0.027 | 0.057 | -0.068 | -0.156 | 0.703 |
| PLT | 5.69E-01 | 1.64E-04 | 1.34E-04 | 6.12E-06 | 1.70E-01 | 9.62E-01 | 5.18E-02 | 1.50E-01 | 1 | 0.028 | -0.232 | 0.111 | 0.107 | -0.088 | -0.115 |
| MPV | 7.47E-03 | 6.57E-02 | 8.85E-02 | 2.46E-01 | 8.88E-16 | 6.25E-12 | 4.05E-01 | 6.60E-02 | 7.05E-01 | 1 | 0.810 | 0.009 | 0.216 | 0.980 | 0.221 |
| PDW | 1.90E-01 | 8.18E-01 | 6.96E-03 | 1.07E-02 | 6.07E-09 | 2.38E-05 | 3.90E-01 | 7.49E-01 | 1.74E-03 | < 1.0E-16 | 1 | 0.005 | 0.123 | 0.866 | 0.261 |
| LYM | < 1.0E-16 | 9.75E-01 | 9.89E-01 | 6.58E-01 | 3.31E-01 | 2.07E-01 | 2.95E-01 | 3.18E-01 | 3.29E-02 | 9.01E-01 | 9.52E-01 | 1 | 0.500 | 0.049 | 0.019 |
| LYMA | < 1.0E-16 | 1.70E-02 | 8.69E-01 | 2.66E-01 | 2.33E-04 | 2.28E-06 | 3.30E-03 | 2.31E-01 | 3.89E-02 | 3.61E-03 | 1.01E-01 | < 1.0E-16 | 1 | 0.266 | 0.051 |
| P-LCR | 2.87E-02 | 1.92E-02 | 2.43E-01 | 5.57E-01 | 1.02E-14 | 1.61E-11 | 3.27E-01 | 6.02E-02 | 2.97E-01 | < 1.0E-16 | < 1.0E-16 | 5.61E-01 | 1.35E-03 | 1 | 0.248 |
| RDW-SD | 3.66E-01 | 1.99E-02 | 3.76E-04 | 1.78E-06 | 1.99E-09 | 4.48E-02 | 5.93E-07 | < 1.0E-16 | 2.47E-02 | 2.69E-03 | 3.65E-04 | 7.17E-01 | 3.25E-01 | 2.60E-03 | 1 |

Above the diagonal for correlation of the 15 hematological traits

Below the diagonal for p-value of correlation in 15 henatological traits
